# LSTM guided homomorphic encryption for threat-resistant IoT networks

**DOI:** 10.1007/s10791-025-09843-4

**Published:** 2025-12-09

**Authors:** Sanjeev Kumar, Sukhvinder Singh Deora, Tajinder Kumar, Purushottam Sharma, Xiaochun Cheng, Vishal Garg

**Affiliations:** 1 Department of Computer Science & Application, Maharshi Dayanand University, Rohtak, India; 2 Department of Computer Science and Engineering, Jai Parkash Mukand Lal Innovative Engineering and Technology Institute, Radaur, Haryana India; 3 School of Computer Science and Engineering, Galgotias University, Greater Noida, Uttar Pradesh India; 4 Computer Science Department, Bay Campus, Swansea University, Fabian Way, Swansea, Wales, SA1 8EN UK

**Keywords:** Internet of things (IoT), Homomorphic encryption (HE), Long short-term memory (LSTM), Anomaly detection, Privacy-preserving computation, Blockchain, Dynamic key management, Multi-factor authentication (MFA)

## Abstract

The rapid growth of the Internet of Things (IoT) has led to revolutionary innovations in many fields; however, it has also resulted in significant security and privacy issues due to the resource limitations and distributed nature of IoT networks. Traditional cryptographic techniques or machine learning-based anomaly detection systems do not jointly provide data privacy and resilience to threats in real time. The existing methods, such as Homomorphic Encryption (HE), offer a high computation cost for performing encryption. Furthermore, Long Short-Term Memory (LSTM) networks can predict an anomaly profile instead of performing encryption. To address these shortcomings, this paper proposes NeuroCrypt. This new hybrid system combines Fully Homomorphic Encryption (FHE) with LSTM-based encrypted anomaly detection and supplements it with blockchain-based dynamic key management and multi-factor authentication. The architecture targets edge and fog computing settings using, among other techniques, ciphertext packing, model quantisation, and parallelised encrypted operations. The performance of the proposed framework has been evaluated on a real dataset. The results show that the accuracy in the proposed framework is 99.2% compared to existing techniques such as HE-based DNN, FL-based models, and LSTM IDS. Conclusively, NeuroCrypt provides a privacy-preserving, effective, and scalable solution to real-time threat abatement in IoT networks.

## Introduction

The short history of our Internet of Things (IoT) has escalated; it encompasses the interactivity of billions of devices, intelligent networks of households, and various other types of diverse wireless devices [1]. This growth has altered many sectors, including the healthcare, transport, manufacturing, agricultural, and energy sectors. The technologies play a significant role in supporting automation, intelligence decision making and distance control, which are essential components of the modern digital economy. Recently, new studies have emphasized that the next-generation internet of things and cyber-physical systems require an integrated security approach, whichentails integrating artificial intelligence and biometrics with encrypted computing. A detailed survey by [2] demonstrates that intelligent networks can be secured with the help of biometric authentication and privacy-saving technologies [3]. In a follow-up paper [4], looked at the open issues in the Industrial-CPS security of the AI age, pointing out the constraints of traditional cryptography in real-time systems. Intelligent decision-making in sensitive domains such as medicine is also performed with deep-learning frameworks, where personalized diagnostics models can perform adaptive, privacy-aware inference [5, 6]. These innovations motivate the creation of hybrid-based architectures such as NeuroCrypt, an amalgamation of encryption, learning, and decentralized trust to scale IoT security. Besides this, serious security and privacy concerns come in conjunction with the revolutionary properties of the IoT ecosystem. The IoT gadgets constitute the resources in their simplest form of purity, and the usual cybersecurity arrangements are the ones that should never be applied to them. The heterogeneous and decentralised nature of the IoT environments supports advanced dynamics.

Concerning the trust management, data confidentiality, and non-disclosure of the various communication protocols and software stack. The alerts of the IoT devices may frequently contain sensitive and internally identifiable information, particularly in fields like healthcare, surveillance, and smart houses, it is vital to ensure that a competent security measure has been, or must be utilized [5]. Conventionally, IoT LeoT ft infrastrat infrastrat geographiostat is protected, which is part of other authenticated systems, e.g. AES or symmetric since asymmetric key establishment recall e.g. RSA, ECC storage gi i.e., stored authentication, which remains to be performed as a delivery or putrefaction progresses. Its methods are generic building blocks of network-layer security protocols (including TLS and internet-exposed service security protocols, including IPSEC [6]). Within the realms of data analytics, there exist data analytics Systems, specifically, the application of signature-based and elementary techniques of machine learning: decision trees, support vector machine (SVM), and random forests, to identify recognized dangers and abnormal behaviors that have already been recognized to have taken place. These systems are often achieved through the application of firewall policy and constant updates of firmware against any control machine-based access processes [7]. However, numerous restrictions exist on using these laid-down security procedures in an IoT. Symmetric encryption involves a shared secret control that is not a scalable one, not even in the terminology of a decentralised topology; asymmetric cryptographic implementations are relatively far more appropriate to the determinant of the distribution of secrets, however, and most certainly never to be implemented by the lightweight key distributing machines [8]. Besides, the older IDS could not keep up, and therefore, could not scale to more complex time dynamic attacks like Advanced Persistent Threat (APT) or zero-day attacks. Even simpler representations of learning might not even be exercised to acquire the chronological interaction or dynamical behaviour of network traffic data on time-series IoT structures; they were not pertinent in acquiring polymorphic malware and protocol-based evasion tactics [9]. A pre-late technique is already predisposed to security against such invasions, which is only enhanced by the advanced anguish chosen in adopting the maturing generation of assaulting methods on the ground of artificial intelligence (AI), record armies, and disseminated assault area. The current high-profile attacks, such as the recent Mirai botnet that installed large numbers of older cameras and routers in a large-scale distributed denial-of-service (DDoS) attack of at least one nature, justify developing secure and robust security models [10]. The number of internet-accessible networks using critical infrastructures, i.e., smart gridding, national transport systems and medical cyberspace networks, helps overpower the loss of money, and even becomes in the future a threat to the survival of society and nations.

### Fully homomorphic encryption (FHE) in IoT

Privacy-preserving computation in Internet of Things (IoT) environments has become especially demanded with the rise in data sensitivity produced by these systems [11]. Healthcare, intelligent surveillance, industrial control systems, and critical infrastructure are some application areas of IoT, which commonly involve personal, behavioural, or proprietary data, the confidentiality of which needs to be maintained across the data lifecycle [12]. Traditional cryptographic systems can secure data at rest and in transit, but fail when it is necessary to decrypt information to process it (such as when performing analytics on the data), putting the data at risk of interception during analysis. Recently, the ability to arbitrarily compute on encrypted data has introduced Fully Homomorphic Encryption (FHE) as a revolutionary technique [13]. This approach provides data confidentiality even in the inference or processing phase, thereby sealing the vulnerability gap exposed by the traditional encryption technique [14].

**Fig. 1:**
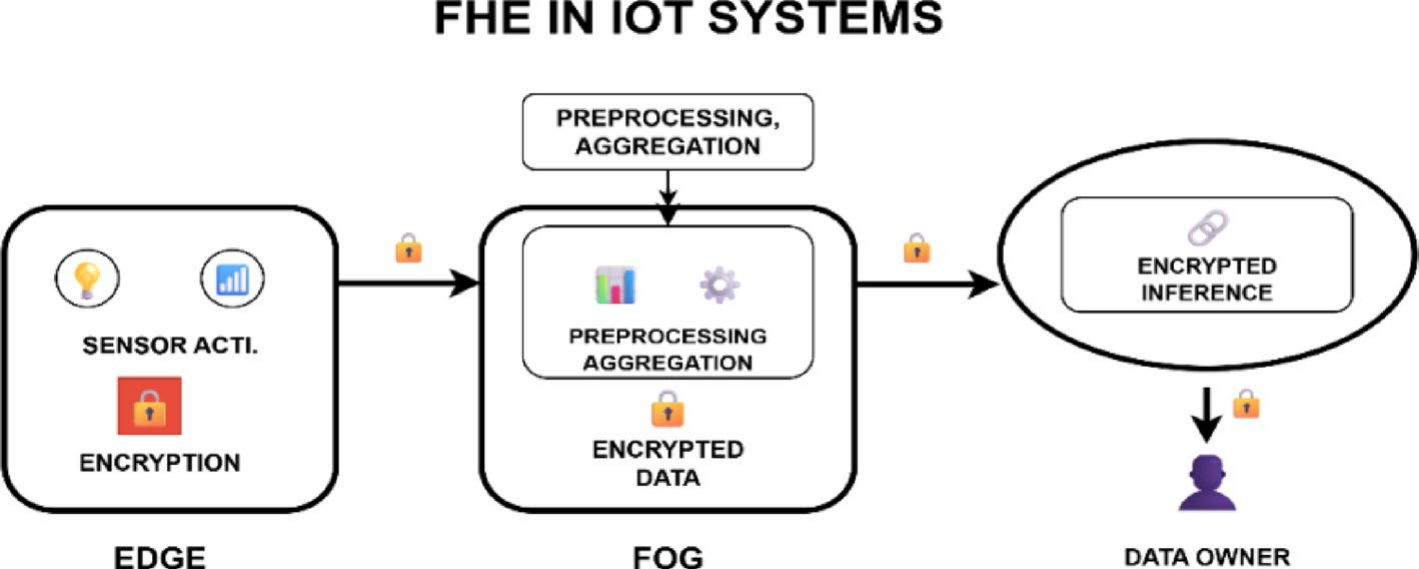
FHE-based IoT systems architecture

Figure 1 depicts the architectural integration of FHE into IoT systems operating across several network layers. Devices that gather raw data about the environment using their sensors and actuators reside at the edge layer [15]. Such devices are often limited regarding memory, power, and computing capacity, meaning that direct FHE computation is out of reach at this layer [16]. However, before sending the collected information, lightweight encryption modules, which can be enhanced by special hardware, can be encrypted using a chosen FHE scheme. Fog/edge servers with greater computation capabilities receive the ciphertext [17].

Furthermore, the fog layer preprocesses the encrypted data. In this case, ciphertext padding techniques that map multiple values to a single ciphertext are applied to allow parallel processing and increase efficiency. The cloud layer receives the encrypted data and conducts more complex and heavy-computation tasks, such as LSTM-based encrypted inference.

Furthermore, FHE will be used at the cloud layer to process the encrypted data. These involve substituting non-linear activation functions with polynomial approximations and weight quantisation to minimise computing depth [18]. Everything is performed in the encrypted space, and the outcome, which remains encrypted, is transmitted back through the identical layers to be lastly decrypted by the data owner. The final encrypted processing system offers extra security for data. At the same time, it’s being used, as well as when it’s being sent or stored, fixing a significant weakness in older IoT security systems.

The benefit of using FHE in IoT systems is that it allows for maintaining data confidentiality without disrupting analytic processes. It enables safe data outsourcing, as it is possible to use third-party analytics platforms or cloud services without losing sensitive data [19]. In addition, FHE can enable the collaborative settings in which two or more parties might be required to compute the common data without revealing their respective contributions. This makes it appropriate for the federated public health or the innovative grid applications of the IoT [20].

### Blockchain for secure and decentralized key management in IoT

The problem of cryptographic key security in a distributed IoT environment is an old and complicated issue. The conventional approach of centralised key management infrastructure or certificate authorities does not apply to IoT networks, as they are decentralised, dynamic, and highly heterogeneous [21]. The devices might regularly enter or abandon the network, not trust one another, and frequently run in an environment with restricted or missing central management. Blockchain technology presents an attractive alternative to decentralised, transparent, and unalterable security credentials management. The permanent record and smart contract support of blockchain technology make it especially well-suited to automate thelifecycle of cryptographic keys, including their generation, distribution, revocation, and assessment [22].

**Fig. 2:**
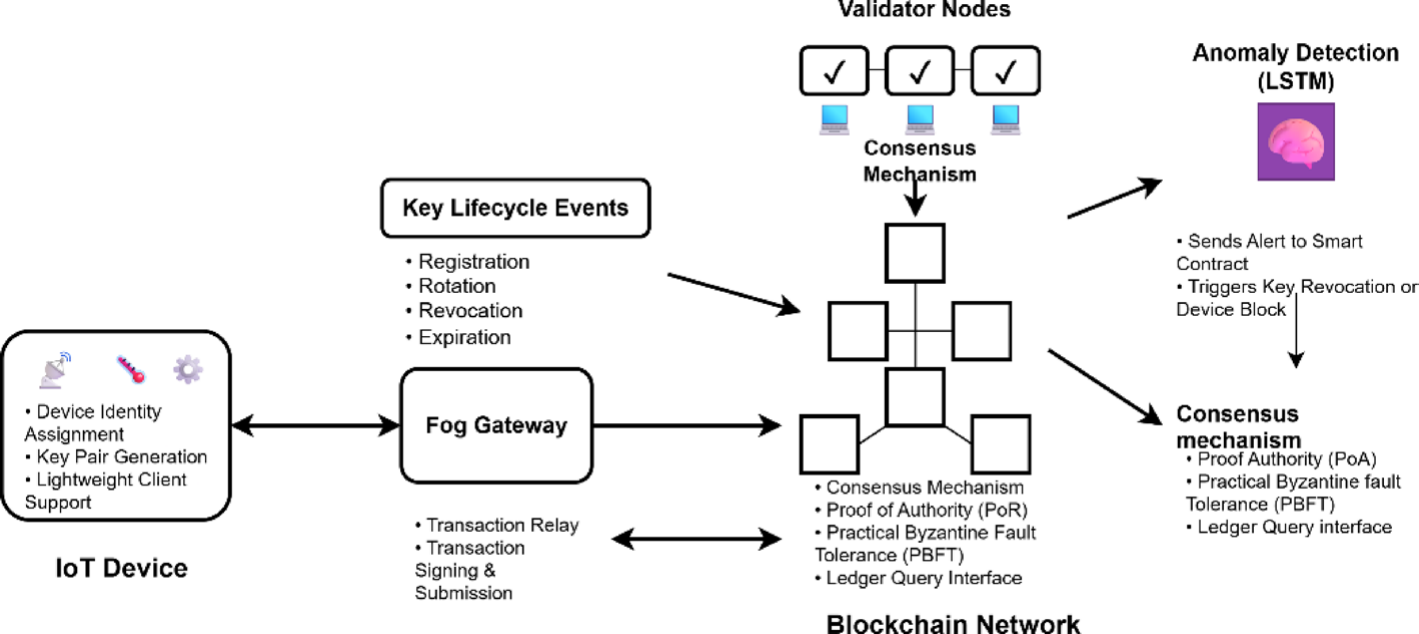
Blockchain-based key management framework in IoT

Figure 2 presents an architecture to implement blockchain into a key management system in IoT, and the architecture is based on distributed registry stored by a set of validator nodes spread all over the network [23]. The consensus protocol allows such nodes to reach an agreement on the current version of the ledger so that updates are verifiable and irreversible. When the device is added to the network, it undergoes a registration transaction signed and transferred to the blockchain (usually by a smart contract). The identity and public key of the device are stored in the ledger [24]. The subsequent lower-level lifecycle events, such as rotation, expiring, or revocation, are also encoded in blockchain transactions.

The magic of this architecture is smart contracts, which are automatically implemented with the logic to enforce the security policies without human involvement. For example, they can be told to carry out automatic key rotations on a schedule, or they can be programmed to automatically revoke keys reported to have been compromised [25]. More crucially, the nature of these contracts is that they can communicate with other components of the system, like anomaly detection modules, to address arising threats. Today, when an LSTM model learns that a device’s behavior is malicious, one can launch a smart contract and revoke the current key of the object, prohibit further communication, and ask the model to reauthenticate with multi-factor credentials. This is done without oversight, and in a permanent manner, and offers a quick and standardized deployment of security obstacles [26].

Clients lacking the means to access the blockchain directly (e.g., because of resource constraints) may also do so through an intermediate node - allowing fog gateways or lightweight clients. Such proxies execute the procedures of creating, signing, and posting transactions as a proxy of limited devices [27]. The decentralized blockchain network may be based on more lightweight consensus schemes, like Proof of Authority or Practical Byzantine Fault Tolerance, so the protocol may still be functional and responsive in low-power and low-bandwidth environments. Any node with the necessary permissions can access a query on the ledger to get any device’s up-to-date credentials and capabilities, thereby sharing trust in the system.

One crucial aspect of the management of IoT is embracing blockchain. It does not need central authorities; thus, single failure points are removed [28]. It is even open in the sense that any modification in the credentials is verifiable publicly by the authorised participants, fostering trust in multi-stakeholder environments. The immutability of the ledger is such that no crucial historical events can be messed with in the future, and this simulates forensics and legal accountability [29]. Moreover, it is possible to program automation of security policies through smart contracts, which can be referred to as a potential way to react to anomalies or system events in real-time and in a data-driven fashion [30].

### LSTM-based anomaly detection in IoT

Firewalls, rule-based filters, and signature-based intrusion detection systems are examples of traditional security systems that are not keeping pace with the dynamism of IoT environments, where new devices, new communication protocols, and new attack vectors are regularly introduced [31]. A powerful alternative is presented by deep learning methods and specifically Long Short-Term Memory (LSTM) networks, which can learn to detect abnormalities in normal behaviour over time, even in cases where the abnormal behaviour is slight or unobserved previously. LSTM models are particularly effective when dealing with sequential information, so they will likely be helpful when presented with time-series inputs produced by IoT devices [32].

**Fig. 3:**
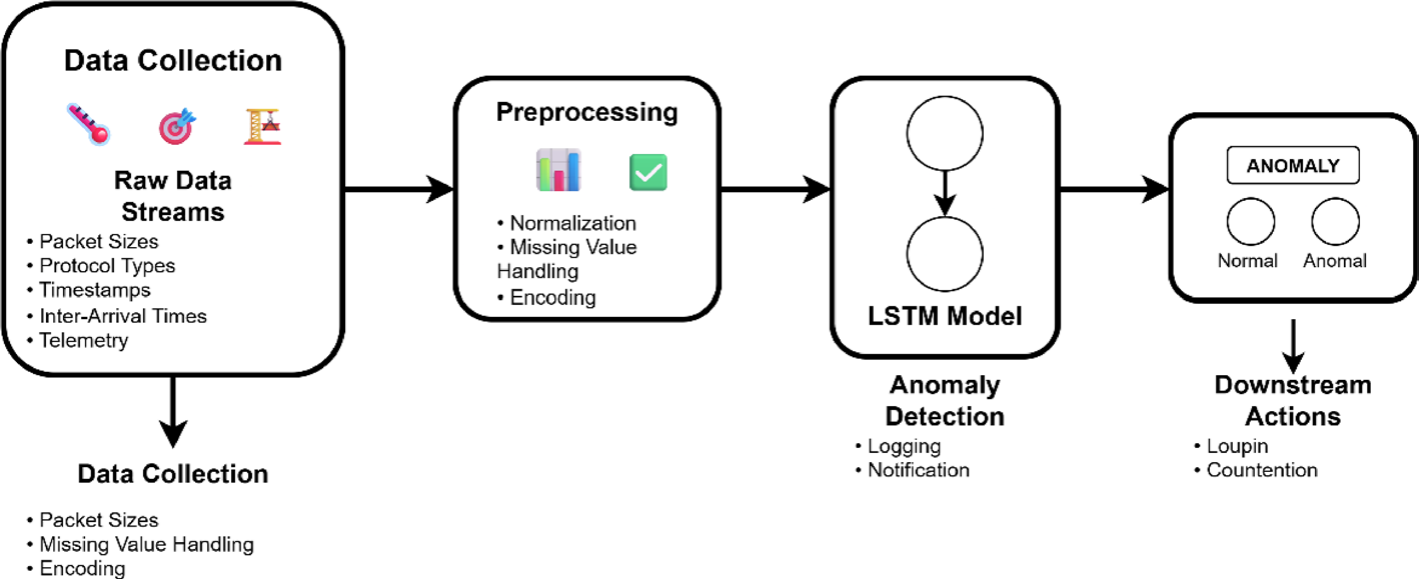
LSTM-based anomaly detection architecture in IoT

Figure 3 shows that an LSTM-based architecture for anomaly detection in IoT starts with the raw data streams gathered by sensors, actuators, or communication interfaces [33]. Features of these data streams are packet sizes and protocol types, timestamps and inter-arrival times, and device-specific telemetry. The data is normalised; missing values are treated, and categorical variables are encoded so that clean and semantic inputs are provided to the model [34]. After the preprocessing, the data is forwarded to the LSTM model, which is usually deployed on fog or cloud computing resources to gain access to more significant computational capabilities.

The LSTM network consists of one or multiple recurrent layers, which employ gating mechanisms to choose what information to keep or discard at each time step [35]. Furthermore, the output retrieved from the LSTM is faded into a dense layer to retrieve the binary classification of anomalies. Anomalies. In specific architectures, autoencoder architectures are employed, where the LSTM is learnt to reconstruct the input sequence, and high reconstruction errors are viewed as anomalies.

Once the anomaly is recognised, it can be recorded for future secure data transmissions [36]. The detection outcome may also be provided as feedback to smart contracts on a blockchain in more sophisticated architectures, allowing access control policies to be enforced in real time and dynamically managed keys. Such close functionality between anomaly detection and trust infrastructure provides a rapid, closed-loop reaction to possible threats.

The advantages of the LSTM-based anomaly detection on IoT are considerable. This is because the model can learn temporal patterns and thus identify advanced threats, which would otherwise slip through rule-based systems. Since it learns from data, the system can be adapted to particular environments and continuously adapted as the behaviour changes. LSTM models can also be trained without access to privacy-sensitive data, as homomorphic encryption or federated learning, and thus can be applied to regulated fields. Furthermore, there are drawbacks, such as the models being computationally expensive and confined to resource-limited devices. Also, the models are black boxes, and explaining decisions and gaining user trust is challenging. Mechanisms to explain the system and rigorous testing will be required to make it reliable and accountable [37].

Comparison Table 1 presents a systematic review of three widely used methods: Fully Homomorphic Encryption (FHE), blockchain-based key management, and LSTM-based anomaly detection in IoT security and privacy. The table presented allows for revealing the complementarity of these technologies by contrasting their advantages and shortcomings in alleviating various aspects of the security and privacy dilemma in an IoT setting. Although FHE, blockchain and LSTM have strengths, none of the methods can comprehensively meet the intricate security requirements of the IoT. FHE ensures data security in processing, but it will not automate trust. Blockchain is also strong in key management, but at the same time, it lacks in behavioral analysis.

**Table 1:** Comparison of the existing techniques for secure data transmission in IoT

Feature/Aspect	Fully homomorphic encryption (FHE)	Blockchain-basedkey management	LSTM-based anomaly Detection
Primary function	Privacy-preserving computation on encrypted data	Decentralized, tamper-proof key management	Real-time anomaly detection via temporal pattern analysis
Main Use Case in IoT	Secure outsourced computation (e.g., cloud-based ML)	Secure lifecycle management of cryptographic keys	Behavioral threat detection in device/network activity
Security Benefit	Data remains encrypted during computation — zero exposure	Eliminates a single point of failure; all events are immutable	Identifies zero-day and adaptive attacks without prior signatures
Architectural Components	Edge devices (encryption), Fog (preprocessing), Cloud (encrypted inference)	IoT devices, Validator nodes, Smart contracts, Ledger	Data collectors, Preprocessors, LSTM models on Fog/Cloud
Key Technologies	Polynomial approximations, Ciphertext packing, Bootstrapping	Distributed ledger, Smart contracts, Consensus protocols	LSTM layers, Autoencoders, Anomaly score classifiers
Device Requirement	Requires offloading due to high computational cost	Lightweight clients or intermediaries for low-power devices	Relies on fog/cloud due to high model complexity
Performance Bottlenecks	High latency and computational overhead, esp. for ML tasks	Scalability and latency in large deployments; smart contract security	Model complexity, latency in offloaded inference, and the need for labeled data
Privacy Support	Strong: computation in the encrypted domain	Moderate: ledger is transparent but doesn’t expose data contents	Moderate to Strong: can be integrated with privacy-preserving ML
Automation Capabilities	None inherently; it depends on the surrounding system	Smart contracts automate policy enforcement and reaction	Can be part of automated threat mitigation when integrated with smart contracts
Challenges	Bootstrapping, parameter tuning, resource constraints	Consensus efficiency, governance, and smart contract vulnerabilities	Model explainability, training data scarcity, black-box nature
Deployment Difficulty	High (requires expert tuning, specialized models)	Medium (requires consensus setup and interface middleware)	Medium to High (requires data pipelines, model training, infrastructure)
Best Fit For	Regulated environments needing secure analytics (e.g., healthcare, smart grid)	Dynamic, decentralized IoT ecosystems need trustless credentialing	Highly dynamic environments prone to novel or stealthy attacks

**Fig. 4:**
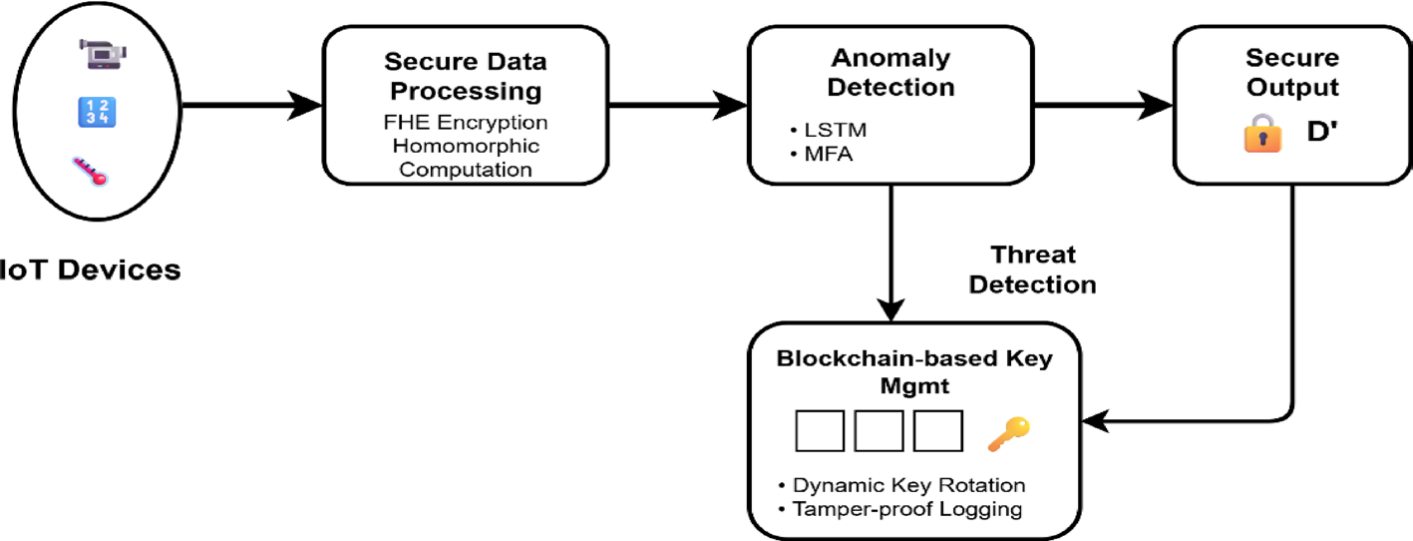
Proposed NeuroCrypt Architecture for secure data transmission

With the architecture depicted in Fig. 4, encrypted traffic can be analyzed using an LSTM model. To apply this in reality, several optimizations are applied in NeuroCrypt. They are packing ciphertext, quantizing the model, parallelizing the running of encrypted operations, and key management via a blockchain. These methods reduce the cost of computing and turn the system into edge and fog devices where computing resources are scarce. This paper will integrate secure data management and innovative and real-time anomaly detection in decentralized IoT networks. NeuroCrypt offers one solution to address privacy and threat detection, a significant step in the next-generation IoT security design. It also facilitates the broader objective of designing AI systems that are reliable, understandable, and scalable in privacy-sensitive and adversarial settings. The work preconditions the future research related to encrypted deep learning, secure edge AI, and federated cybersecurity structures. NeuroCrypt is designed on five interrelated elements: the Edge Device, the Fog Gateway, the Cloud-side Homomorphic Encryption (HE) Nodes, the Blockchain Layer, and the Key Management Service. All the components operate using a well-defined data domain, plaintext or ciphertext, to ensure a high level of performance and confidentiality. The edge employs lightweight AES-256 when doing regular flows and only encrypts with HE when directed by the Guidance Module. The HE scheme does not store private keys. It receives pre-processed plaintext features and outputs an anomaly score. $\:{P}_{anom}\in\:\left[\text{0,1}\right]$. When $\:{P}_{anom}\ge\:\theta\:$, it requests an HE public key via a blockchain smart contract and encrypts the subsequent window of packets. The fog, therefore, determines when homomorphic evaluation will be conducted, weighing the cost of privacy and latency. Homomorphic computation of encrypted data is processed. They receive ciphertext inputs. $\:{c}_{i}=\text{Enc}({x}_{i},p{k}_{t})$and evaluate model functions $\:{f}_{HE}$Using CKKS arithmetic. Only authorized owners can decrypt the resulting ciphertexts. The key issuance and revocation of smart contracts is recorded in the system as hash, signature, and events. Smart contracts apply crucial lifetimes and can initiate multi-factor authentication or re-training models on abnormal notifications. Generates short-lived key pairs $\:(p{k}_{t},s{k}_{t})$for each HE session, publishes the public key to the blockchain, and ensures private keys remain with the data owner.

**Table 2:** Description of artifacts and their domains in the neurocrypt framework

Artifact	Examplefields	Domain	Owner / who stores it	Purpose
Raw packet features	timestamp, pkt_size, flags, inter-arrival	Plaintext at edge; ephemeral at fog	Edge device → fog preprocessing buffer	Input to LSTM Guidance Module
LSTM anomaly score P_anom	scalar ∈ [0,1]	Plaintext at fog	Fog (local), logged summary on blockchain	Guidance decision
Selected packets window	n packets after alert	Ciphertext (FHE) when protected; AES otherwise	Encrypted and sent to cloud HE nodes	Homomorphic inference
HE session public key	key_pk_t	plaintext metadata on blockchain	KeyMgmt / blockchain	Enables the cloud to evaluate the ciphertext
HE result	encrypted inference output	Ciphertext until decrypted by the authorized party	Cloud → returned to owner	Final decision, decrypted by the owner

Table 2 presents the key artifacts of NeuroCrypt, including the description of sample fields, each a plaintext or ciphertext, its owner, and the purpose.

**Algorithm 1:**
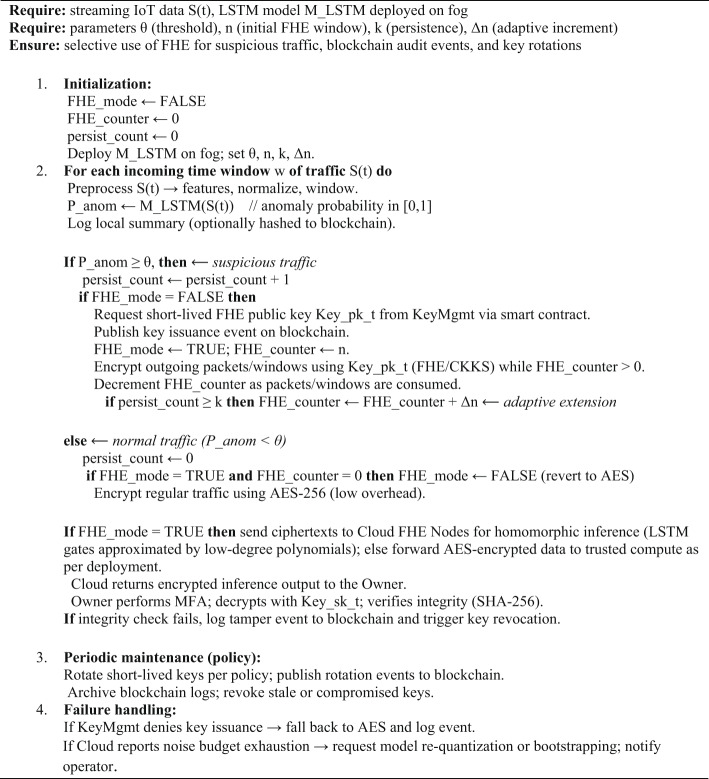
LSTM-Guided Selective FHE Processing (NeuroCrypt)

LSTM only operates on plaintext and is only used on the fog layer in the NeuroCrypt framework. It examines lightly filtered momentary IoT information and provides a probability score of an anomaly. Local AES 256-encryption protects the brief exposure of plaintext when the data is sent or stored in buffers. Once the score falls below a predefined threshold, it is encrypted with Fully Homomorphic Encryption (FHE) and sent to the cloud to be encrypted and inferred. Since the typical LSTM models do not support encrypted numbers, the cloud node computes the secure computations using polynomially approximated LSTM gates. Such a setup has helped to defend the sensitive traffic, and on the other hand, the fog can easily identify irregularities.

## Literature review

The recent works have become more focused on the question of how to integrate different kinds of deep learning, specifically the Long Short-Term Memory (LSTM) network, with a variety of cryptographic mechanisms (such as Homomorphic Encryption (HE)) and fuzzier machine learning (FL) approaches to safeguard information throughout its lifecycle. These hybrid models can (1) remove the capability of more sophisticated cyber threats since they can learn temporal and contextual dependencies and (2) guarantee privacy owing to the encrypted computation. The versatility of different contributions exploring secure neural models, HE-friendly architectures, blockchain-based key management, and privacy-preserving approaches to learning is observed in the literature investigating the generic contexts of the IoT ecosystems. Recent research has sought to develop efficient and sustainable deep learning systems, which enhance accuracy in the face of limited data and computing resources. In addition to encryption-related studies, several researchers have examined sustainable and optimized deep-learning solutions to address data shortage and resource limitations in IoT security. Senanayake et al. [38] provided an approach through which multiple organisations may employ machine learning models without revealing their confidential information to each other. They utilize the SMPC, which enables them to undertake the required calculations on encrypted data and, simultaneously, maintain the privacy of inputs. Decentralised counter-terrorism needs all sites; all sites are identical, and personal information is guaranteed on both ends.

Manh et al. [39] proposed a privacy-enabled framework for identifying cyber threats in IoT-enabled blockchain networks. Safe sharing of IoT data with a cloud service provider uses the CKKS scheme and a SIMD algorithm. Experimental results have been presented that indicate that the proposed technique can provide 91% accuracy with minimal overhead, which is nearly the same as the non-encrypted performance.

Badawi et al. [40] introduced OpenFHE, which was motivated by earlier FHE libraries, including PALISADE, HElib and HEAAN. OpenFHE supports bootstrapping and changing schemes and can provide hardware acceleration via a Hardware Abstraction Layer. It has modes specific to users and compilers to meet different development needs. The information about the architecture is provided in the current document, and additional documents can be explored individually.

Lee et al. [41] proposed ResNet-20 with the RNS-CKKS FHE scheme with bootstrapping and tested it on the CIFAR-10 data. They did not choose to replace ReLU and Softmax with simple alternatives, but used sophisticated techniques to evaluate their action well. Indeed, they have verified that deep learning using private-key encryption is feasible, as they achieved 92% accuracy, close to that obtained by a non-encrypted model.

Gentry [42] proposed a fully homomorphic encryption (FHE) framework that allows one to evaluate any function on encrypted data. The point is that a fully homomorphic encryption can run its own optimised decryption circuit on encrypted input – a condition referred to as bootstrap. Due to this effort, the contemporary FHE systems could evolve.

Cheon et al. [43] depict that with their scheme, it is possible to perform addition and multiplication on encoded real numbers. A significant development is when a process is used to maintain precision, and noise can remain low when the modulus is varied. They can do encrypted math using a special batching technique, RLWE, and cyclotomic rings, which do not reduce accuracy.

Bhandari. R [44] investigated how various deep learning techniques preserve privacy, focusing on key issues, penetration testing events and resistance measures. Differential privacy and homomorphic encryption typically address model inversion attacks. The review helps to understand that, despite the development of valuable tools, federated learning and secure data management should be used continuously in all ML processes to protect privacy fully.

Narkedimilli et al. [45] suggested a type of federated learning (FL) designed for the Internet of Things (IoT) that uses different advanced security features to meet privacy and security needs. The framework allows the use of Decentralised Attribute-Based Encryption (DABE), local data encryption, and decentralised authentication in IoT. HE enables individuals to operate on encrypted information securely, and SMPC enables the training of machine learning models without sharing confidential information. Due to blockchain, SMPC gives its result securely and offers an open explanation of changes and the integrity of all information in the FL network.

Bossuat et al. [46] provided recommendations of parameter sets in Learning With Errors (LWE) to facilitate their application in Fully Homomorphic Encryption (FHE) schemes over various levels of security. They determine a broad spectrum of FHE parameter configurations regarding the degree of effectiveness of each and the level of protection. Besides that, they also examined available open-source tools that assist in parameter selection, contributing to thefeasibility of how FHE is employed in practice.

Xie et al. [47] discussed and reviewed techniques that improve HE in PPFL. The review covers general optimisation, algorithmic techniques, hardware and hybrid optimisation techniques and examines their issues and challenges. A mapping of optimisation methods is provided, making recommendations for future work to enable larger and practical HE-based PPFL systems.

Liu et al. [48] divided the literature on homomorphic encryption into three groups based on the development generated by the extension of PHE to AHE: bootstrapping, precision improvement, and solution enhancement. The paper categorises AHE use cases into groups according to the complexity of the application. It outlines the most recent trends, providing insights into the possible future of research and utilisation of AHE.

Asynchronous federated learning was proposed by Xiong et al. [49] to ensure that privacy remains preserved even in edge-based environments with multimedia data. In their approach, they rely on RABE and DP to guarantee complete privacy and flexibility in participation across devices. Experimental evaluation of the scheme indicates that its run time reduces by 63.3% in cryptography, 61.9% in global aggregation, and it is highly accurate on MNIST (86.7%), CIFAR-10 (70.8%) and Fashion-MNIST (86.1%).

Istaltofa et al. [50] trained linear regression and LSTM models on the price data of Bitcoins obtained on Yahoo Finance from 2014 to 2024. The findings state that LSTM is much more adequate than linear regression, resulting in lower MSE and RMSE and a perfect R² performance. LSTM seems to be particularly effective in dealing with sudden changes observed in cryptocurrency, providing an advantage in financial trend prediction.

Xiang et al. [51] reviewed the applicability of LSTM, neural networks and CNN to predict prices in the cryptocurrency industry. To improve the predictive ability of the models, the study employs technical indicators and sentiment data. The best precision was observed when the three architectures were used in a hybrid model, as witnessed by the RMSE value of BTC, ETH and BNB. Irrespective of these concerns, this paper identifies why deep learning-based hybrid models could help enhance the accuracy of forecasts and support risk management in digital asset markets.

They experimented with various new models to test how well the deep learning approaches could predict the price of cryptocurrencies, as reported by Wu et al. [52]. The authors tested variations of LSTM networks, CNNs and the Transformer model. Univariate and multivariate tools were examined based on various cryptocurrencies to predict close prices a few weeks into the future. A volatility analysis proved that, in the context of the COVID-19 pandemic, the prices underwent substantial changes quickly. Two separate tests were conducted: (1) based on pre-COVID-19 data, how well the models would estimate the initial period of the pandemic and (2) based on new COVID-19 data, to predict prices in future years. Convolutional LSTM generated the highest prediction accuracy when the methodology utilised several input variables.

Singla. S [53] suggested a method to achieve security in the message exchange between devices in the Industrial Internet of Things (IIoT) with the help of Spiking Neural Networks (SNNs) and blockchain. Calculations with SNN’s consumption are much quicker and use less memory. If the information on the IIoT network is not malicious, blockchain seals it to prevent malicious individuals from altering it. Combining the Interplanetary File System (IPFS) and smart contracts can vastly improve the effectiveness and speed at which data can be used and processed. 5G enhances the architecture, thus reducing latency and improving reliable network packets. They determine the effectiveness and security of the system by quantifying various parameters, like loss, different time scales, contract performance, and the bit error rate (BER).

Kumar and Kim [54] suggest identifying cyberattacks in the Internet of Health Things (IoHT) with the help of both FL and LSTM networks. EFL-LSTM implies that FL preserves sensitive information, and LSTM can capture time-series activities that can assist in identifying cyber threats. The fact that feature selection is implemented in-house makes the system more efficient, and FL ensures that the process can be extended and run in a decentralised manner.

Jony et al. [55] present a concept of an LSTM-based IDS system that detects both existing and new types of cyber threats. The findings emphasise the model as flexible and precise, which offers what is required for next-generation security solutions to cope with emerging patterns of attacks. The problem of IoT security is raised in the work, and a solution is introduced that can be applied to different systems.

Sarkar et al. [56] explained why RNNs and a form of vector-valued neural synchronisation could assist in the safe exchange of keys in the IIoT. The approach is based on drive-response mechanics that enhance synchronisation and cryptography in IIoT scenarios requiring tight timing. The study focuses on the architecture of coupled RNNs as they react to different input and output delay types. Besides this, the work also considers response-based RNN systems without delays, since this direction of secure neural-based communication protocols that can be applied to IIoT devices has been scarcely studied.

Prasanna et al. [57] introduced a method to identify network threats depending on how events and sequences of network traffic correlate on different time scales using LSTMs—the operations aid in reducing the false positives and increasing the system accuracy. Placing BDA alongside the LSTM model, the researchers addressed the issues connected to the complexity of calculation and training, and the interpretation of the model became significantly faster. The suggested IDS was deployed on Apache Spark with the NSL-KDD dataset and performed effectively against various parameters, demonstrating superior performance to the conventional IDS methods.

Despite the advances in providing IoT with better security via privacy-preserving computation and intelligent anomaly detection, there is still a huge gap in research at the newly emerging aggregation of encrypted deep learning and real-time and adaptive threat mitigation on resource-constrained IoT devices. Table 3 shows a comparison of existing techniques used in the literature. There have been previous studies on all permutations of homomorphic encryption (HE), secured multiparty computation (SMPC), federated learning (FL), and blockchain to protect sensitive data, and it has been shown that LSTM and deep neural models are effective at modelling the temporal dynamics of cyberattacks. These methods, however, tend to focus on either privacy or detection accuracy alone and rarely simultaneously without significant latency, scalability, or implementation complexity trade-offs. The current HE-based systems are usually limited in adaptability and computationally expensive. In contrast, the LSTM-based systems, despite their accuracy, need access to plaintext and therefore cannot support strong privacy guarantees. Moreover, although research considers FL or differential privacy in a decentralised learning environment, few have considered deploying these mechanisms into a coherent framework that can perform encrypted model inference, allow dynamic key management, and achieve regulatory compliance (e.g., GDPR). As a result, an integrated solution capable of secure, low-latency, and intelligent analysis of encrypted IoT data is required, which is currently not sufficiently examined.

**Table 3:** Comparison of the existing techniques

Ref No.	Contributions	Techniques	Dataset	Results	Research Gap	Limitations
[38]	SMPC-based encrypted ML across institutions	SMPC, CNN, Logistic Regression	Structural & functional MRI	Secure multi-party computation with practical ML models	No real-time threat modeling	High computational cost, limited temporal adaptability
[39]	Privacy-preserving threat detection for blockchain IoT	CKKS, SIMD, DNN, Distributed Learning	IoT-based blockchain dataset	91% accuracy with minimal overhead	Limited adaptability to evolving threats	No integration with LSTM or time-series analysis
[40]	OpenFHE: open-source FHE library with hardware acceleration	FHE, HAL, PALISADE, HEAAN	Library and framework-level evaluation	Support for bootstrapping and compiler-level development	No integration with intelligent models or IoT-specific scenarios	Generic platform, lacks use-case-specific implementations
[41]	HE with deep learning (ResNet) for encrypted image classification	RNS-CKKS, ResNet-20	CIFAR-10	92% accuracy under encryption	No anomaly detection or real-time capability	Focused on classification, not security context
[42]	First design of Fully Homomorphic Encryption (FHE)	Ideal Lattices, Bootstrap technique	Theoretical framework	Proof-of-concept for FHE feasibility	Not optimized for real-time or applied ML tasks	High computational complexity, non-practical early model
[43]	Approximate HE arithmetic with low-noise real number encoding	CKKS, RLWE, Cyclotomic Rings	Mathematical validation	Efficient encrypted math with batching and low noise	No use in intelligent anomaly detection frameworks	No ML integration or adaptive learning
[44]	Penetration testing framework for deep learning privacy tools	Differential Privacy, HE, FL	Review and analysis framework	Highlighted privacy vulnerabilities and mitigation techniques	No concrete model implementation or integration strategy	Conceptual review, lacks empirical results
[45]	FL with blockchain and DABE for IoT	FL, SMPC, DABE, Blockchain	Simulated IoTframework	Secure FL training with decentralized key management	No deep learning-based anomaly detection	Complex key management, lacks encrypted inference
[46]	Security guidelines and parameter sets for FHE	LWE, Open-source tools	Cryptographic parameter simulations	Practical recommendations for FHE use in real systems	No AI model or time-series analysis integration	Parameter design focused, not application-oriented
[47]	Optimization strategies for HE in PPFL	HE, Federated Learning, Hardware Optimization	Literature survey	Mapped challenges and solutions for large-scale HE systems	No implementation or evaluation in IoT networks	Survey-based, lacks experimental verification
[48]	Survey on Approximate HE (AHE) and its evolution	AHE, CKKS, Bootstrapping	Comparative review	Identified trends and use-case categorizations	No model or framework-based implementation	Focused on theoretical progression
[49]	Asynchronous FL for multimedia in edge IoT	RABE, Differential Privacy, FL	MNIST, CIFAR-10, Fashion-MNIST	High accuracy with reduced runtime and global aggregation	No homomorphic encryption or encrypted learning integration	Focus on FL, limited to multimedia
[50]	Compared LSTM and Linear Regression on Bitcoin data	LSTM, Linear Regression	Yahoo Finance (BTC 2014 to 2024)	LSTM outperformed regression with lower MSE and RMSE	No security context or encrypted data handling	Application-specific, not security-driven
[51]	Hybrid DL model with LSTM, CNN, NN for crypto forecasting	LSTM, CNN, Neural Network	BTC, ETH, BNB	Hybrid model improved forecasting accuracy	No privacy-preserving or encrypted analytics used	Focused on finance, lacks real-time constraints
[52]	Evaluated LSTM, CNN, Transformer for crypto prediction	LSTM, CNN, Transformer	Pre- and Post-COVID cryptocurrency datasets	Conv-LSTM achieved highest accuracy on multivariate inputs	No encrypted framework or threat-resilience tested	Financial context, no cyberthreat modeling
[53]	Secure IIoT messaging using SNN and Blockchain	SNN, Blockchain, IPFS, Smart Contracts	IIoT network simulation	Low latency, tamper-proof communication	No deep temporal learning or encrypted AI	No LSTM or predictive anomaly detection
[54]	Cyberattack detection in IoHT using FL and LSTM	FL, LSTM, Feature Selection	ECU-IoHT	Better than traditional models, protected data	No encrypted model inference	Limited integration with HE or secure computation
[55]	LSTM-based IDS for cyber threat detection	LSTM	CIC-IoT2023	98.75% accuracy, F1-score 98.59%	No privacy-preserving mechanisms	Operates on plaintext, not encrypted data
[56]	RNN-guided neural synchronization for IIoT key exchange	Coupled RNNs, Drive-Response Mechanism	IIoT communication framework	Secure key exchange protocol with delay analysis	No encrypted analytics or anomaly detection	Protocol-focused, lacks detection layer
[57]	Big data-aware LSTM IDS with reduced false positives	LSTM, BDA, Apache Spark	NSL-KDD	Better detection than traditional IDS	No encryption or secure computation	Privacy concerns, lacks secure key management

**Fig. 5:**
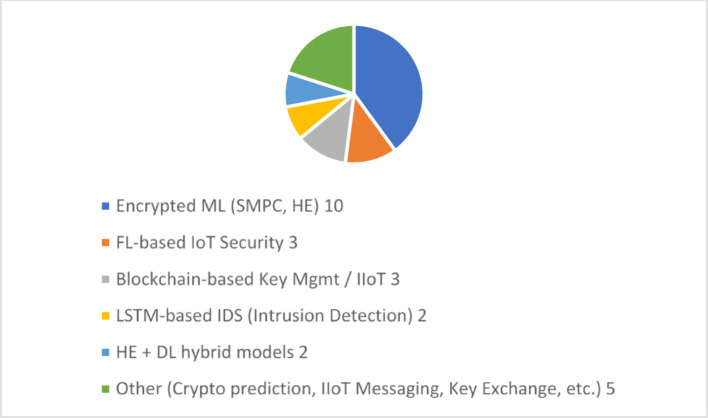
Classification of the techniques used in literature

Figure 5 shows the existing literature’s techniques for secure and privacy-preserving IoT networks. The surveyed works also concentrate much on encrypted machine learning (40%) (SMPC, HE), which has attracted much attention recently in the desire to perform computation on encrypted data. The remainder of the literature, 10%, is FL-based IoT Security and Blockchain-based Key Management, both focusing on making decentralised trust and privacy-preserving federated learning a reality. LSTM-based Intrusion Detection Systems (IDS) and HE + Deep Learning hybrid models follow with 6–7% each, a small, although growing, use of temporal deep learning methods with encryption. The remaining contributions, 17%, are categorised into various areas, including crypto prediction, IoT messaging, and key exchange protocols. This analysis shows that while there have been significant developments in different research areas like HE or FL, very few existing studies combine encrypted anomaly detection, adaptive key management, and blockchain-based trust, highlighting the uniqueness of the proposed NeuroCrypt framework.

## Problem statement

Although both encrypted computation and deep learning-based intrusion detection have enormous potential, the existing IoT security frameworks lack an integrated solution that guarantees real-time threat detection, end-to-end data privacy, and scalability. The majority of the current solutions consider privacy and intelligence as orthogonal objectives: Cryptographic techniques keep data safe when it’s stored or being sent, but to analyse it, the data has to be decrypted, which can expose sensitive information during the process; LSTM-based models are excellent at spotting unusual patterns over time, but since they work with unencrypted data, they can’t be used in sensitive areas like healthcare or critical infrastructure.

A promising candidate is Fully Homomorphic Encryption (FHE), which promises to allow computations on encrypted data. Its use is, however, delayed by the high computational expense, complicated model updates, and inefficiency in real-time applications, particularly when used with deep learning models such as LSTM. In addition, secure key management is also a bottleneck. The centralised IoT system is not suited to be decentralised and dynamic in structure and character, and the blockchain-based solutions, despite being more optimistic, are hardly ever combined with encrypted analytics and intelligent threat response seamlessly.

Most systems use fixed threat models and cannot rotate keys, reconfigure policies, or otherwise update detection models in reaction to new attack patterns. IoT surroundings are at a constant risk of emerging and advanced threats without real-time flexibility.

In such a way, the central issue is the absence of a coherent framework that would enable encrypted anomaly detection, decentralised trust, and real-time flexibility without performance losses. Ideal research should incorporate the cryptographic power of FHE, the predictive power of LSTM, and the decentralised trustworthiness of blockchain to realise secure, scalable, and intelligent threat remedies. NeuroCrypt fills this gap with a proposal of a hybrid architecture with the capabilities of encrypted inference, dynamic key management, and tamper-evident logging, a new paradigm of privacy-preserving IoT security.

## Proposed methodology

To address the complicated issues discussed earlier in a complete way, this section introduces NeuroCrypt. This system uses Fully Homomorphic Encryption (FHE), LSTM-based anomaly detection, and blockchain-based key management to ensure secure and smart threat reduction in IoT networks. NeuroCrypt offers encrypted data processing and real-time threat detection in the same architecture, unlike traditional solutions, where privacy and analytics are addressed differently. It is tuned to edge, fog, and cloud deployments via model quantisation and ciphertext packing, among others, to guarantee low latency and minimal resource utilisation. The framework also allows dynamic and decentralised key management and strong access control through the integration of smart contracts and multi-factor authentication, rendering it scalable and able to meet contemporary data protection regulations.

**Fig. 6:**
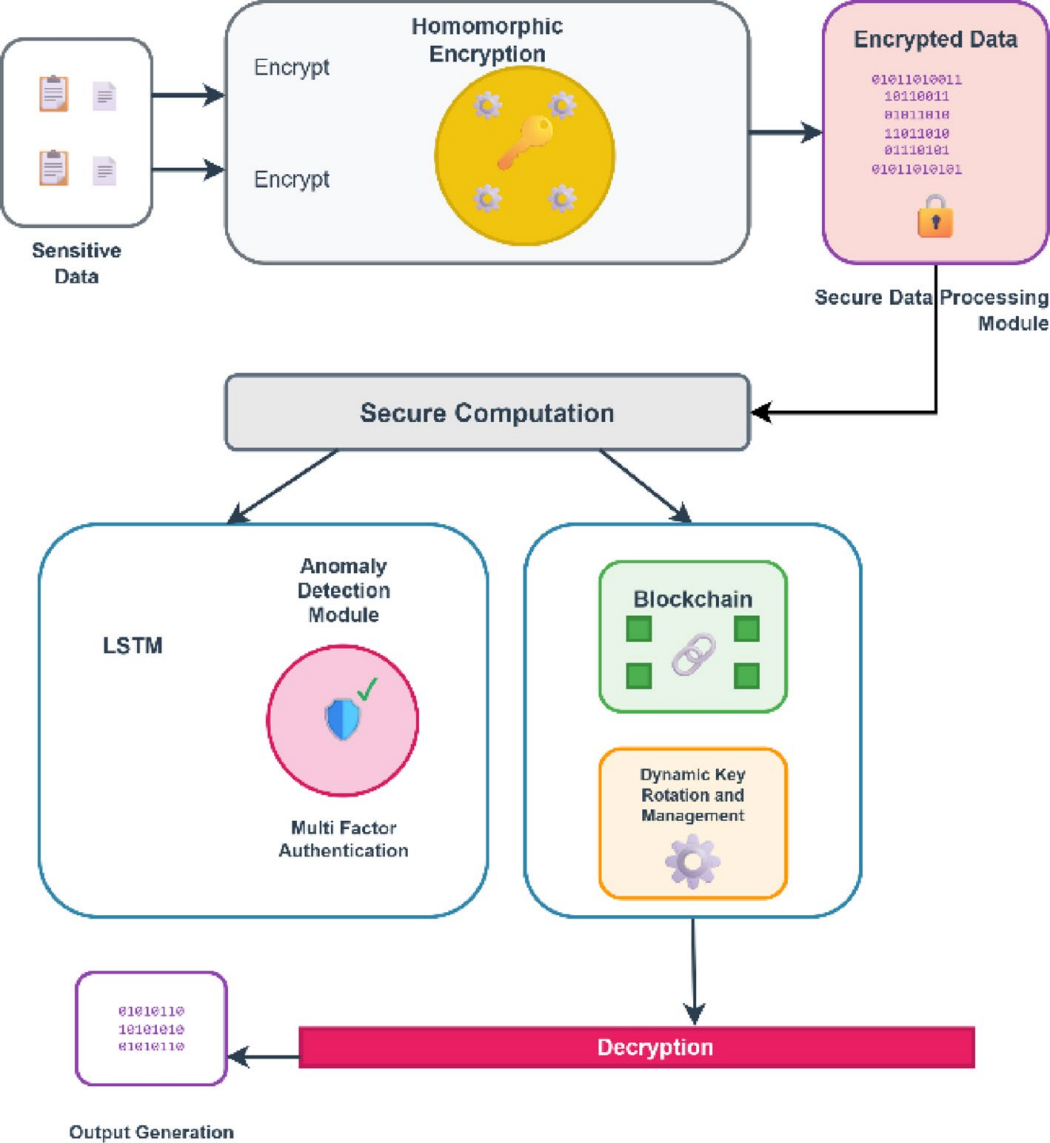
Working of the proposed NeuroCrypt architecture

Figure 6 shows the architecture of the proposed NeuroCrypt framework, which consists of fully homomorphic encryption (FHE), LSTM-based anomaly detection, blockchain-based dynamic key management, and multi-factor authentication (MFA) to realise privacy-preserving and dynamic threat detection in IoT networks. The initial stage is the encryption of the sensitive IoT data with homomorphic encryption, which enables the secure computation of ciphertext without revealing the raw data. The ciphertext is further submitted to an anomaly detection module, where an LSTM model can learn the temporal patterns and identify the possible threat. Access control to the anomaly detection pipeline is additionally enforced using MFA mechanisms. This system constantly communicates with a blockchain layer, a dynamic key rotation, and a management component to make sure that encryption keys are safely rotated and all security-related events are logged permanently, which provides tamper-evident auditing.

**Algorithm 2:**
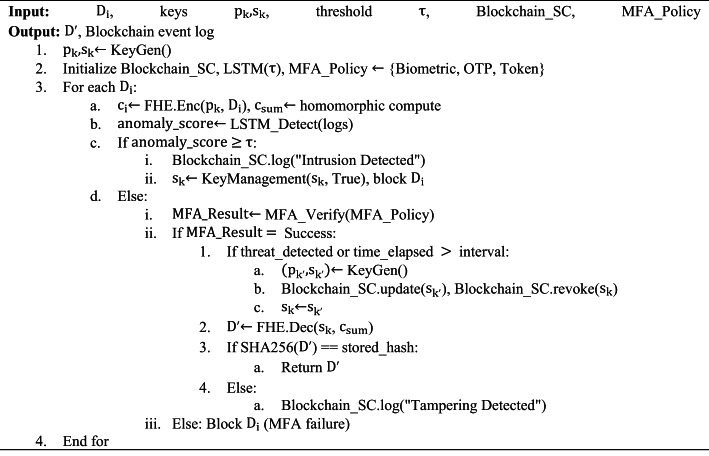
Secure IoT Data Processing with FHE, Blockchain, and MFA

**Fig. 7:**
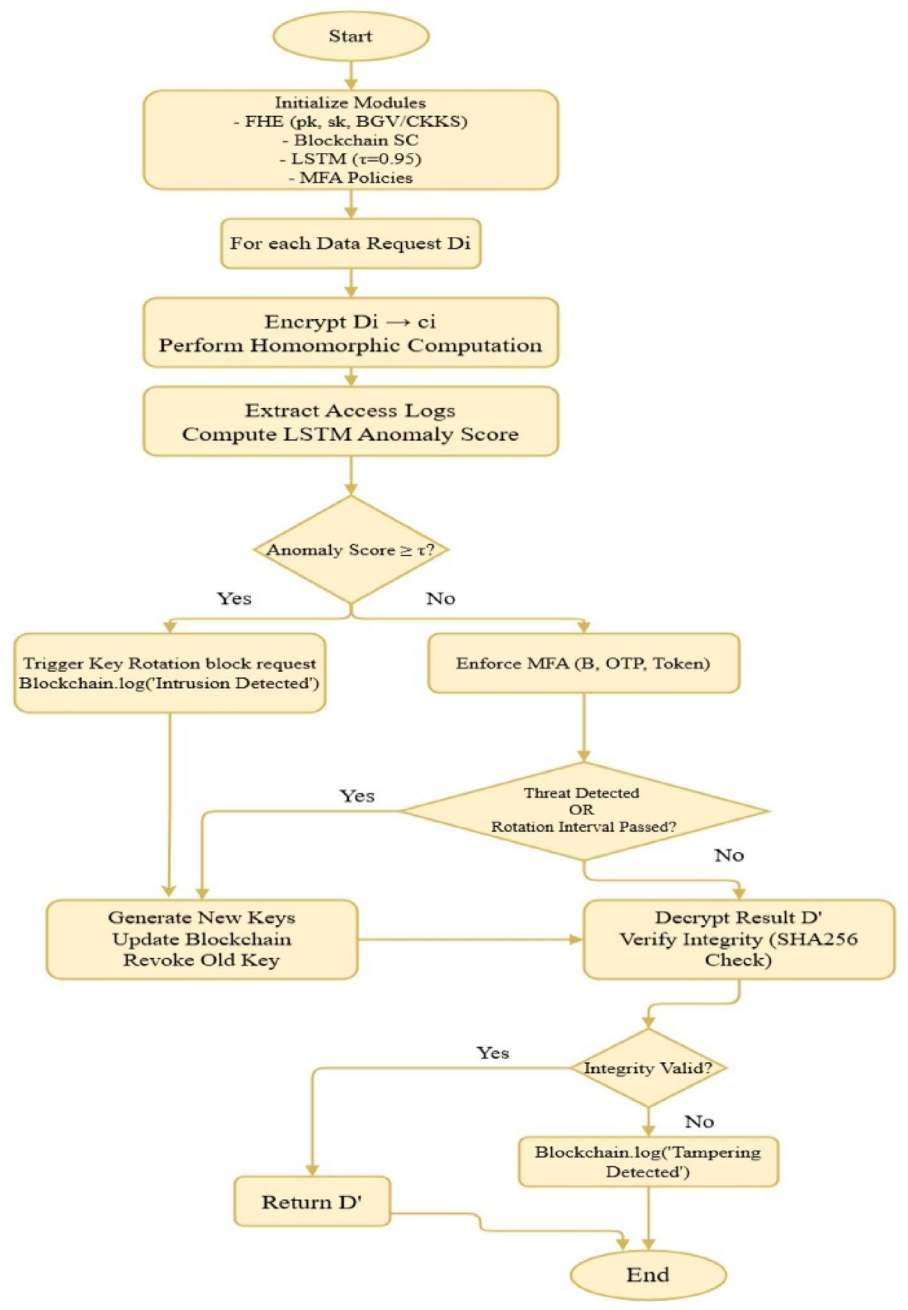
Flowchart for the processing of NeuroCrypt

Figure 7 depicts the flowchart of the complete work process of the AHE-BKM algorithm of secure and intelligent processing of IoT data. The workflow is initialised by preparing such major modules as Fully Homomorphic Encryption (FHE), Blockchain Smart Contracts (SC), LSTM anomaly detection models, and Multi-Factor Authentication (MFA) policies. On each incoming data request $\:{\text{D}}_{\text{I}}$, the data is encrypted to a ciphertext $\:{\text{c}}_{\text{i}}$, and homomorphic computation is performed on it. Access logs are next extracted, and an LSTM-based anomaly score is estimated. A dynamic key rotation is initiated by the system in case the anomaly score exceeds a preconfigured threshold $\:T$, the request is blocked, and an intrusion entry is recorded on the blockchain. MFA is applied in case of no detected anomaly, and the system assesses if a threat is detected or if a key rotation period has expired. During either a threat or a rotation scenario, new encryption keys are issued, the blockchain is updated, and old keys are cancelled. At this point, the ciphered output is deciphered, and integrity is checked with SHA256 hashing.

### LSTM guidance module for encrypted anomaly detection

The most critical advancement of the NeuroCrypt framework is the integration of Fully Homomorphic Encryption (FHE) with Long Short-Term Memory (LSTM) anomaly detection. FHE ensures high privacy, yet it is computationally expensive, which renders it impractical to implement on all the IoT traffic. The module takes the output of the probability of anomaly of the LSTM. It converts it into a transparent encryption choice to ensure the sensitive traffic is not compromised, but does not overload resource-constrained devices.

**Fig. 8:**
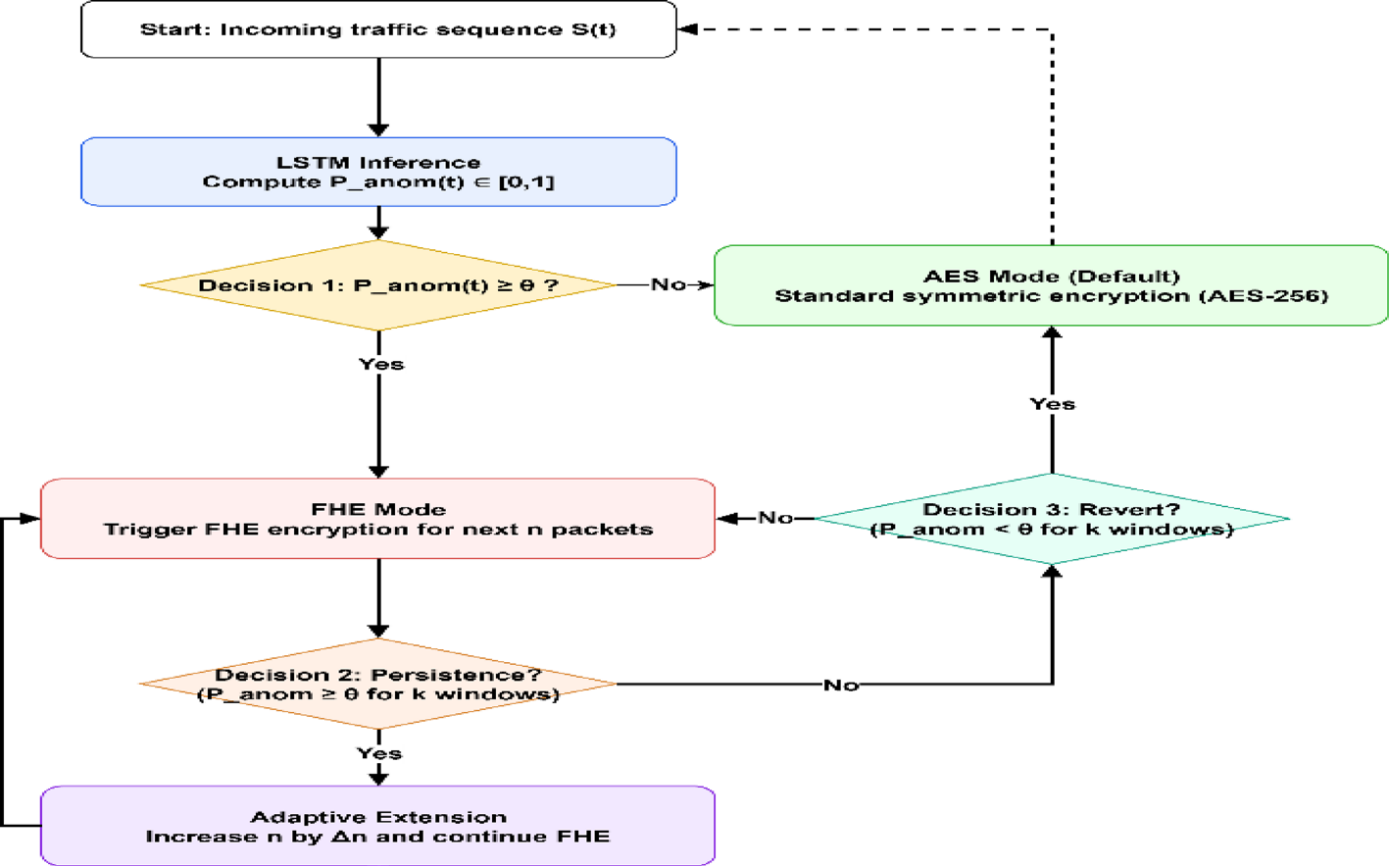
Flowchart of the LSTM Guidance Module

The proposed LSTM Guidance Module presented in Fig. 8 used a fixed anomaly-detection threshold of 0.95, which was a conservative design, and such a design guarantees that only flows with an extremely high probability of an anomaly activate FHE encryption. This environment is more security-conscious by reducing false negatives, which is the highest risk of allowing malicious traffic to make it through without being encrypted. When activated, the module encrypts the subsequent *n* = 50 packets, which is approximately one second of IoT traffic sufficient to cover burst anomalies, but not excessively using resources. In these conditions, protection against threats is scaled, and an adaptive increase of 25 packets by the encryption window is gradually increased.

**Algorithm 3:**
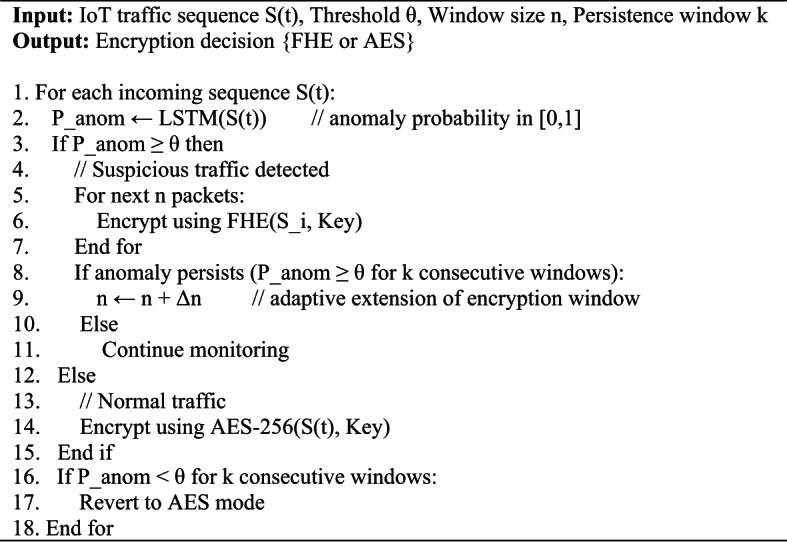
LSTM-Guided Encrypted Processing

The parameters that govern this process are determined through careful design considerations. The threshold θ acts as the decision boundary for switching between AES and FHE modes. The window size n specifies the number of packets to be encrypted with the FHE, following the detection of an anomaly by ensuring short bursts of suspicious traffic are adequately covered. The persistence window k provides stability by requiring anomalies to continue across multiple observation windows before the adaptively extending FHE coverage, thereby avoiding unnecessary reactions to these transient spikes. Finally, in the adaptive increment, Δn defines how much of the FHE coverage is extended when anomalies persist, enabling the system to scale its protection according to the severity and duration of the threat. These parameters ensure that the LSTM Guidance Module balances strong privacy with computational efficiency in real-time IoT environments.

### Proposed mathematical model

This section is organised in a way that gradually establishes the data flow, computation procedures, and system security assurances.

#### System definition

Here, the NeuroCrypt system is abstracted as a mathematical tuple of different modules: data input, an encryption and decryption mechanism, a machine learning model, key management, a blockchain ledger, and an authentication system. This abstract representation lays the foundation for modelling secure computation over IoT data. All of the elements of the tuple have their particular role, ensuring the general integrity and privacy of the framework.

The NeuroCrypt system is defined as a tuple:

1 $$\:\mathcal{N}=(\mathcal{D},\mathcal{E},\mathcal{D}\text{e}\text{c},{\mathcal{M}}_{{\uptheta\:}},{\mathcal{K}}_{\text{t}},\mathcal{B},\mathcal{A},{\uptau\:})$$

where:

- $\:\mathcal{D}$ is the IoT data stream $\:\{{\text{d}}_{1},{\text{d}}_{2},\dots\:,{\text{d}}_{\text{T}}\}$.
- $\:\mathcal{E}$ is the Fully Homomorphic Encryption (FHE) function: $\:\mathcal{E}:{\mathbb{R}}^{\text{n}}\times\:{\mathcal{K}}_{\text{t}}\to\:{\mathbb{C}}^{\text{n}}$.
- $\:\mathcal{D}\text{e}\text{c}$ is the corresponding FHE decryption function.
- $\:{\mathcal{M}}_{{\uptheta\:}}$ is the encrypted LSTM anomaly detection model with parameters $\:{\uptheta\:}$.
- $\:{\mathcal{K}}_{\text{t}}=(\text{p}{\text{k}}_{\text{t}},\text{s}{\text{k}}_{\text{t}})$ denotes the public/private key pair at time $\:\text{t}$.
- $\:\mathcal{B}$ is a blockchain ledger used to store cryptographic events and key rotations.
- $\:\mathcal{A}$ is the Multi-Factor Authentication mechanism.
- $\:{\uptau\:}$ is the anomaly detection threshold.

#### Data encryption and transformation

In this case, we outline the encryption of IoT data, as it has not yet been analyzed. This is why Fully Homomorphic Encryption (FHE) is applied to every data vector and what qualities it should have to be used in secure computation. The transformation guarantees that the raw data will never be revealed in the processing, and downstream tasks, like anomaly detection, can be carried out without confidentiality issues.

Each data vector $\:{\text{d}}_{\text{t}}\in\:{\mathbb{R}}^{\text{n}}$ is encrypted as:

2 $$\:{\text{c}}_{\text{t}}=\mathcal{E}({\text{d}}_{\text{t}},\text{p}{\text{k}}_{\text{t}})$$

where $\:{\text{c}}_{\text{t}}$ is the ciphertext. This transformation preserves the semantic security of $\:{\text{d}}_{\text{t}}$, even when used for computation.

Homomorphic operations on ciphertexts obey:

3 $$\:\mathcal{E}\left({\text{d}}_{1}\right)\oplus\:\mathcal{E}\left({\text{d}}_{2}\right)=\mathcal{E}({\text{d}}_{1}+{\text{d}}_{2}),\:\mathcal{E}\left({\text{d}}_{1}\right)\otimes\:\mathcal{E}\left({\text{d}}_{2}\right)=\mathcal{E}({\text{d}}_{1}\cdot\:{\text{d}}_{2})$$

These properties allow encrypted input to be processed directly without exposing raw features.

#### Encrypted LSTM processing

This concerns implementing the encrypted data into a specially adapted LSTM model. Because the conventional LSTM operations are non-linear and cannot be used with homomorphic encryption, they are represented by low-degree polynomials. This adaptation enables the model to be applied securely on ciphertexts and allows recognition of time-dependent patterns on encrypted streams, with no information leakage, as explained in the section.

In the proposed framework, the non-linear activation functions of the LSTM gates (sigmoid and tanh) are approximated using third-degree Chebyshev polynomials. We adopt this method because Fully Homomorphic Encryption (FHE) cannot evaluate several exponential or hyperbolic functions efficiently. Cubic approximation provides a good tradeoff between viability and precision. Lower-degree polynomials do not fit the curvature of the activation functions and add more serious approximation errors. In contrast, higher-degree polynomials add more multiplicative depth to encrypted operations, which results in too high latency. This demonstrates that the polynomial replacement makes encrypted inference feasible without compromising anomaly detection effectiveness.

Let an LSTM cell have input. $\:{x}_{t}$, hidden state $\:{h}_{t-1}$, and cell state $\:{c}_{t-1}$. The LSTM gates in plaintext are:

4 $$\:\begin{array}{c}{f}_{t}=\sigma\:({W}_{f}{x}_{t}+{U}_{f}{h}_{t-1}+{b}_{f})\\\:{i}_{t}=\sigma\:({W}_{i}{x}_{t}+{U}_{i}{h}_{t-1}+{b}_{i})\\\:{\stackrel{\sim}{c}}_{t}=tanh({W}_{c}{x}_{t}+{U}_{c}{h}_{t-1}+{b}_{c})\\\:{c}_{t}={f}_{t}\odot\:{c}_{t-1}+{i}_{t}\odot\:{\stackrel{\sim}{c}}_{t}\\\:{o}_{t}=\sigma\:({W}_{o}{x}_{t}+{U}_{o}{h}_{t-1}+{b}_{o})\\\:{h}_{t}={o}_{t}\odot\:tanh\left({c}_{t}\right)\end{array}$$

In NeuroCrypt, all these functions are approximated with low-degree polynomials (e.g., Chebyshev or Taylor approximations):

5 $$\:\sigma\:\left(x\right)\approx\:\stackrel{\sim}{\sigma\:}\left(x\right),\:tanh\left(x\right)\approx\:\stackrel{\sim}{tanh}\left(x\right)$$

Encrypted LSTM output becomes:

6 $$\:{\widehat{y}}_{t}={\mathcal{M}}_{\theta\:}^{enc}\left({c}_{t}\right)$$

where $\:{\mathcal{M}}_{\theta\:}^{enc}$ Applies all operations homomorphically.

#### Anomaly detection and key management

Following the encrypted inference step, the framework uses a thresholding operation to identify potential outliers. This describes how an observed anomaly results in constructing another key pair, and a blockchain ledger is modified. By so doing, the system ensures reactive and proactive security control, which guarantees against active threats and exposure in case of a breach. The Practical Byzantine Fault Tolerance (PBFT) consensus protocol runs in the blockchain layer (herein referred to as blockchain) of the proposed NeuroCrypt framework. PBFT has been chosen instead of resource-consuming authentication, like Proof of Work, due to its lightweightness, low energy use, and applicability to permissioned IoT. A blockchain network was set up, and the validator nodes were deployed on fog and cloud computing servers to avoid extra computing power charges to the constrained edge devices. Such a design allows unreliable validation of key management transactions: generation, rotation, and revocation processes can be realized without excessive load on the IoT devices. The resilience offered by the selection of PBFT also provides resilience to a fraction of malicious or faulty validators and has low communication and computation overhead. It measured the consensus performance of transaction throughput and latency, which are essential to support near real-time key updates and logging of anomalies in the IoT networks.

Anomaly score $\:{\widehat{y}}_{t}$ Is compared against a detection threshold $\:\tau\:$:

7 $$\:{\text{Flag}}_{t}=\mathbb{I}[{\widehat{y}}_{t}>\tau\:]$$

If $\:{\text{Flag}}_{t}=1$, dynamic key rotation is triggered:

8 $$\:{\mathcal{K}}_{t+1}=\text{KeyGen}\left(\right),\:\mathcal{B}.\text{append}({\mathcal{K}}_{t+1},t)$$

The blockchain ledger $\:\mathcal{B}$ is implemented as:

9 $$\:{\mathcal{B}}_{i}=\text{Hash}({\mathcal{B}}_{i-1}\parallel\:{\text{KeyEvent}}_{i}\parallel\:{\text{Sig}}_{i})$$

Each new key event is digitally signed and linked via cryptographic hashes to ensure immutability and auditability.

#### Multi-factor authentication and decryption

After a threat has been analyzed, its decrypted findings are protected by a Multi-Factor Authentication (MFA) system. This is the process of authentication that integrates user identity, token authentication, and biometric authentication.

To authorize decryption:

10 $$\:{\mathcal{A}}_{t}=\text{MFA}(user,token,biometric)$$

If $$\:{\mathcal{A}}_{t}=\text{True}$$, then:

11 $$\:{{d}^{{\prime\:}}}_{t}=\mathcal{D}ec({\widehat{y}}_{t},s{k}_{t})$$

This ensures only authenticated users have access to decrypted results.

#### Security definitions

This section formalizes the security guarantees provided by the NeuroCrypt framework. It proposes four main properties, i.e., data confidentiality, model confidentiality, blockchain integrity, and end-to-end privacy. Each of them is characterized by probability boundaries to describe the resilience of security against different adversaries. These definitions are the marks that the system has to fulfill to be declared secure.

Data Confidentiality:

12 $$ \:\forall \:{\mathcal{A}}:Pr[{\mathcal{A}}(c_{t} ) \Rightarrow \:d_{t} ] \le \: \in ,\:{\text{(negligible)}} $$

Model Confidentiality

13 $$\:Pr\left[\mathcal{A}\right({\widehat{y}}_{t})\Rightarrow\:\theta\:]\le\:\delta$$

Blockchain Integrity:

14 $$\:\text{Tamper}\left(\mathcal{B}\right)\Rightarrow\:\text{Collision}\left(\text{Hash}\right)\vee\:\text{Break}\left(\text{Sig}\right)\Rightarrow\:\text{negligible}$$

End-to-End Privacy:

15 $$\:\text{Leakage}\left({\mathcal{M}}_{\theta\:}^{enc}\right({c}_{t}\left)\right)\approx\:0$$

#### Theorems and lemmas

This proves the key security theorem of the system, which confirms that the joint application of FHE, blockchain, and MFA ensures solid defense against adversarial threats. Theoretical assumptions in its support include semantic security and unforgeability. The theorem summarises the key NeuroCrypt model claim: that it can enable privacy-preserving, tamper-resistant, and auditable inference over encrypted IoT data.

##### Theorem 1

Privacy-Preserving Threat-Resilient Inference.

Under the assumptions of FHE semantic security, digital signature unforgeability, collision-resistant hashes, and enforced MFA, the system guarantees all defined properties for any polynomial-time adversary $\:\mathcal{A}$.

#### Supporting lemmas

To prove the theorem, this presents the necessary lemmas for why each component acts safely within specified limits. These comprise the viability of assessing LSTM functionalities in a homomorphic way, the verifiability of the blockchain entries, and the impossibility of leakage of inferences due to the encryption of the intermediate results. These lemmas give the logical spine that supports the main theorem.

##### Lemma 1

Homomorphic LSTM Evaluation.

Under the assumption that each non-linear activation in LSTM can be estimated with a degree d polynomial, then:

16 $$\:{\mathcal{M}}_{\theta\:}^{enc}\left({c}_{t}\right)\in\:\text{HE-Supported\:Operations}$$

##### Lemma 2

Blockchain Verifiability.

If $\:{\mathcal{B}}_{\text{i}}=\text{Hash}({\mathcal{B}}_{\text{i}-1}\parallel\:{\text{Event}}_{\text{i}})$, then altering $\:{\mathcal{B}}_{\text{j}}$ for $\:\text{j}<\text{i}$ requires recomputing valid hash chains, which is computationally infeasible.

##### Lemma 3

Encrypted Privacy.

Since all intermediate states of $\:{\mathcal{M}}_{{\uptheta\:}}$ are encrypted and never exposed:

17 $$\:\text{P}\text{r}\left[\mathcal{A}\right(\text{view})\Rightarrow\:{\text{d}}_{\text{t}}]\le\:\text{negl}\left({\uplambda\:}\right)$$

9. Unified Equation Pipeline.

This section gives an overview of the whole operation pipeline of NeuroCrypt. Information is transferred between inputs and outputs by a series of steps: encryption, encrypted inference, anomaly flagging, key management and conditional decryption. The following steps are presented in a linear order to explain the role played by each mathematical operation in making the system reliable.

18 $$\:{\text{d}}_{\text{t}}\stackrel{\mathcal{E}}{\to\:}{\text{c}}_{\text{t}}\stackrel{{\mathcal{M}}_{{\uptheta\:}}^{\text{e}\text{n}\text{c}}}{\to\:}{\widehat{\text{y}}}_{\text{t}}\stackrel{\text{Threshold}}{\to\:}{\text{Flag}}_{\text{t}}\stackrel{\text{KeyGen,\:MFA,\:Dec}}{\to\:}{{\text{d}}^{{\prime\:}}}_{\text{t}}$$

10. Final Guarantee.

The conclusion proves the overall security status of the NeuroCrypt framework. This conclusion summarizes the model and indicates it is effective in real-life Internet of Things security situations.

For all $\:\text{t}$ in execution time horizon $\:\text{T}$ and all adversaries $\:\mathcal{A}$ bounded by $\:\text{poly}\left({\uplambda\:}\right)$, the NeuroCrypt framework ensures:

19 $$\:\text{Confidentiality}\wedge\:\text{Integrity}\wedge\:\text{Adaptivity}\wedge\:\text{Auditability}$$

## Results and discussion

This section provides the results of the proposed NeuroCrypt framework’s performance metrics of detection accuracy, computational overhead, latency, privacy preservation effectiveness, and scalability. As done in previous studies, simulated IoT network traffic datasets with injected anomalies were used to evaluate it. The objective is to formalise that NeuroCrypt achieves real-time, privacy-preserving, and adaptive threat detection under resource limitations characteristic of IoT edges and fog conditions.

### Dataset used

To evaluate the performance of the proposed framework, we considered an extensive IoT network traffic dataset to test the efficiency of the suggested NeuroCrypt framework, which covers normal device operations and a broad spectrum of cyberattacks. The data is a collection of labelled traffic flows produced by numerous IoT devices in an innovative environment, including smart home hubs, surveillance cameras, smart locks, bright lights, and IoT sensors. The data consists of benign and malicious traffic, including Distributed Denial-of-Service (DDoS) attacks, botnet communication, spoofing, injection attacks, malware traffic, and reconnaissance. The data has been obtained in IoT network environments realistically, meaning the traffic patterns represent how modern IoT devices behave and their vulnerabilities. The dataset’s characteristics of every traffic flow are denoted as a sequence of features based on packet-level and flow-level statistics. These attributes extract significant temporal and behavioural aspects of the network traffic, which is essential for effectively identifying anomalies using the LSTM-based model in NeuroCrypt. The dataset was preprocessed before training the models, and the steps involved were data cleaning, normalisation, and division into time-series sequences with a window of 50 time steps, as that is the structure of input that the LSTM architectureexpects. The processed data consists of an equal mixture of regular and malicious activity, facilitating robust training and testing of the proposed threat detection system. Table 4 gives a summary of the significant attributes utilised in the dataset.

**Table 4:** Description of attributes in the dataset

Attribute Name	Description
Timestamp	Time of packet or flow observation
Source IP Address	IP address of the sending device
Destination IP Address	IP address of the receiving device
Source Port	Network port used by the source device
Destination Port	Network port used by the destination device
Protocol	Network protocol used (TCP, UDP, ICMP, etc.)
Packet Count	Total number of packets in the flow
Packet Size (Bytes)	Size of individual packets or total flow size
Flow Duration (ms)	Duration of the network flow
Inter-Arrival Time (ms)	Time between consecutive packets
Flags	TCP flag indicators (e.g., SYN, ACK, FIN)
Payload Size	Size of the payload within the packet
Anomaly Label	Label indicating whether the traffic is benign or malicious (binary label)

Table 5 provides a comprehensive overview of the CIC-IoT2023 dataset. Approximately 16.7% of the flows represent benign traffic, such as DNS, HTTP, and MQTT communications from smart home and industrial IoT devices, while the remaining 83.3% consist of malicious flows covering a broad spectrum of injected attack scenarios. These include high-volume Distributed Denial of Service (DDoS) and Denial of Service (DoS) flooding attacks, brute force attempts on authentication services such as SSH and FTP, Mirai-like botnet communications, reconnaissance and scanning activities, and malware or injection traffic.

**Table 5:** CIC-IoT2023 dataset statistics and features

Category	Count	Percentage	Notes
Total Flows	33,000,000+	100%	IoT traffic collected from smart home/industrial IoT devices
Benign Flows	~ 5,500,000	~ 16.7%	Normal traffic (DNS, HTTP, MQTT, etc.)
Malicious Flows	~ 27,500,000	~ 83.3%	Multiple attack scenarios injected
DDoS/DoS	~ 12,000,000	~ 36%	High-rate flooding (UDP/TCP/HTTP-based)
Brute Force / Password	~ 3,200,000	~ 9.7%	SSH/FTP brute force
Botnet / Mirai-like	~ 4,500,000	~ 13.6%	Botnet traffic from compromised IoT nodes
Reconnaissance/Scan	~ 3,800,000	~ 11.5%	Port scanning, service probing
Injection/Malware	~ 4,000,000	~ 12.1%	Code injection, malware payloads
Spoofing/Man-in-Middle	> 1000+	~ 0.002%	Limited representation
Features Extracted	80+	~ 0.0001%	Includes packet-level, flow-level & time-series statistics (see below)

The dataset was first put through a structured preprocessing pipeline, including data cleaning, normalization, and sequence preparation, before being made available to the training process. When cleaning the data, repeated flows and incomplete records were eliminated to ensure consistency. Your missing values were addressed by imputation: without affecting your training, continuous numbers-related features were filled by median, categorical variables by their mode, and records with more than 20% missing items were dropped to eliminate the presence of noise. After the data was cleaned, it was normalized to scale all features to a similar level. Min-max normalization of continuous variables was calculated, where words of the range [0,1], attributes like packet size (length in bytes), and flow duration could not control the learning process. One-hot encoding decodes categorical variables into a machine-interpretable format, such as network protocols and TCP flags. This made each feature contribute to the training and enhanced model convergence fairly. Lastly, the dataset was split into time-series sequences that can be processed with LSTM-based anomaly detection. Flows on the network were separated into sliding windows with 50 time steps, so the modeling could study the temporal dynamics of the traffic pattern, but not rely on the individual packets or flows. Each sequence was assigned a label using majority voting, where it would be labeled malicious if most of its constituent flows are labeled malicious and benign otherwise.

### Performance metrics

We utilized a variety of performance metrics to thoroughly assess the efficiency of the NeuroCrypt framework. These measures evaluated the capacity of the system to identify anomalies more precisely, maintain low computationalcosts, and provide real-time reactions and safeguard the privacy of information when implementing encrypted computations.

#### Detection accuracy

Detection accuracy is a ratio of correctly classified examples in the dataset, both benign and malicious traffic. The high accuracy value shows that the LSTM-based anomaly detector is suitable for detecting attacks without generating too many false positives or false negatives.

20 $$\:\text{Accuracy}=\frac{\text{T}\text{P}+\text{T}\text{N}}{\text{T}\text{P}+\text{T}\text{N}+\text{F}\text{P}+\text{F}\text{N}}$$

Where:

- TP = True Positives (malicious correctly detected).
- TN = True Negatives (benign correctly detected).
- FP = False Positives (benign incorrectly flagged as malicious).
- FN = False Negatives (malicious traffic missed).

#### Inference latency

The inference latency is the duration required to process one sequence of encrypted IoT traffic with the LSTM model and obtain a detection result. This phase is one of the most important steps to ensure that this system can provide real-time or near-real-time threat detection even on resource-limited IoT devices.

#### Computational overhead

The three key measures utilised in estimating the computational overhead in NeuroCrypt include the CPU usage, the memory usage, and the time taken during encryption and decryption. All these measures are used to identify the efficiency of the system and the number of resources it needs during the work. CPU usage measures processing overheads due to cryptographic computations, whereas memory consumption indicates RAM usage during data processing and key handling. Data transformation and retrieval speed can be attained with the time required to encrypt and decrypt information.

#### Key management performance

Since dynamic key management is an inherent feature of NeuroCrypt, the latter is also included in the analysis concerning its efficiency, which is measured by two significant performance indices. First is key rotation latency, or the delay implied in rotating encryption keys periodically or when required. This is an essential step towards availing a feature to the system to change cryptography keys based on security policies or other threats in a timely fashion without disrupting other processes. The second is the blockchain transaction latency, i.e., the time required to log important management events, e.g., generation, rotation, or revocation, and to prove related cryptographic keys on the blockchain.

### Results

This section outlines the outcomes of the NeuroCrypt framework, and its efficiency is discussed in terms of the two frequent issues: protection of privacy and threat detection in IoT networks in real time. Our performance metrics to measure the framework are accuracy in detection, latency in inference, computing cost, and efficiency in key management. Our results indicate that NeuroCrypt has better security and scalability than existing algorithms such as HE + DNN, FL -DABE -BC, and LSTM IDS.

**Fig. 9:**
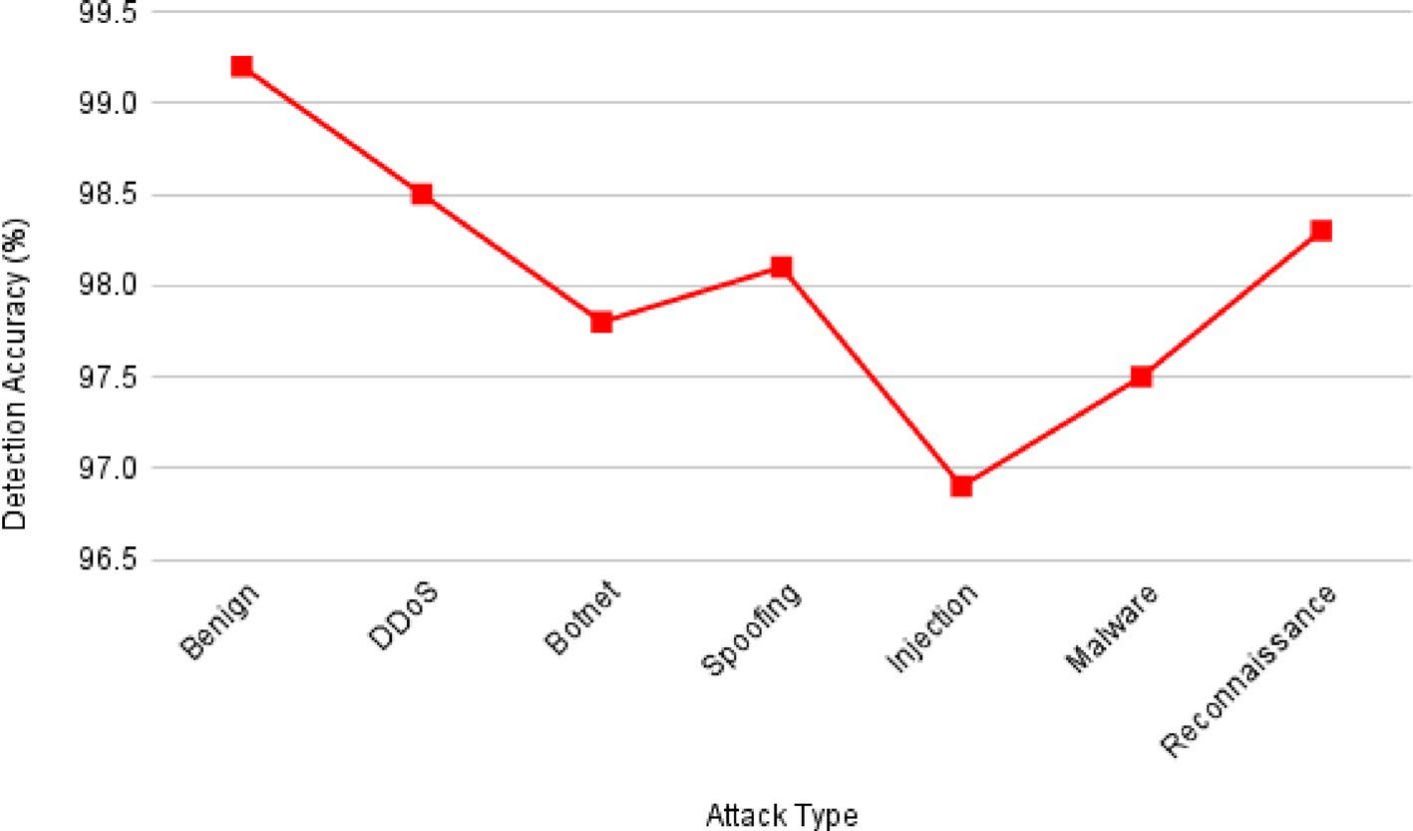
NeuroCrypt Detection accuracy for different classes of attacks

**Fig. 10:**
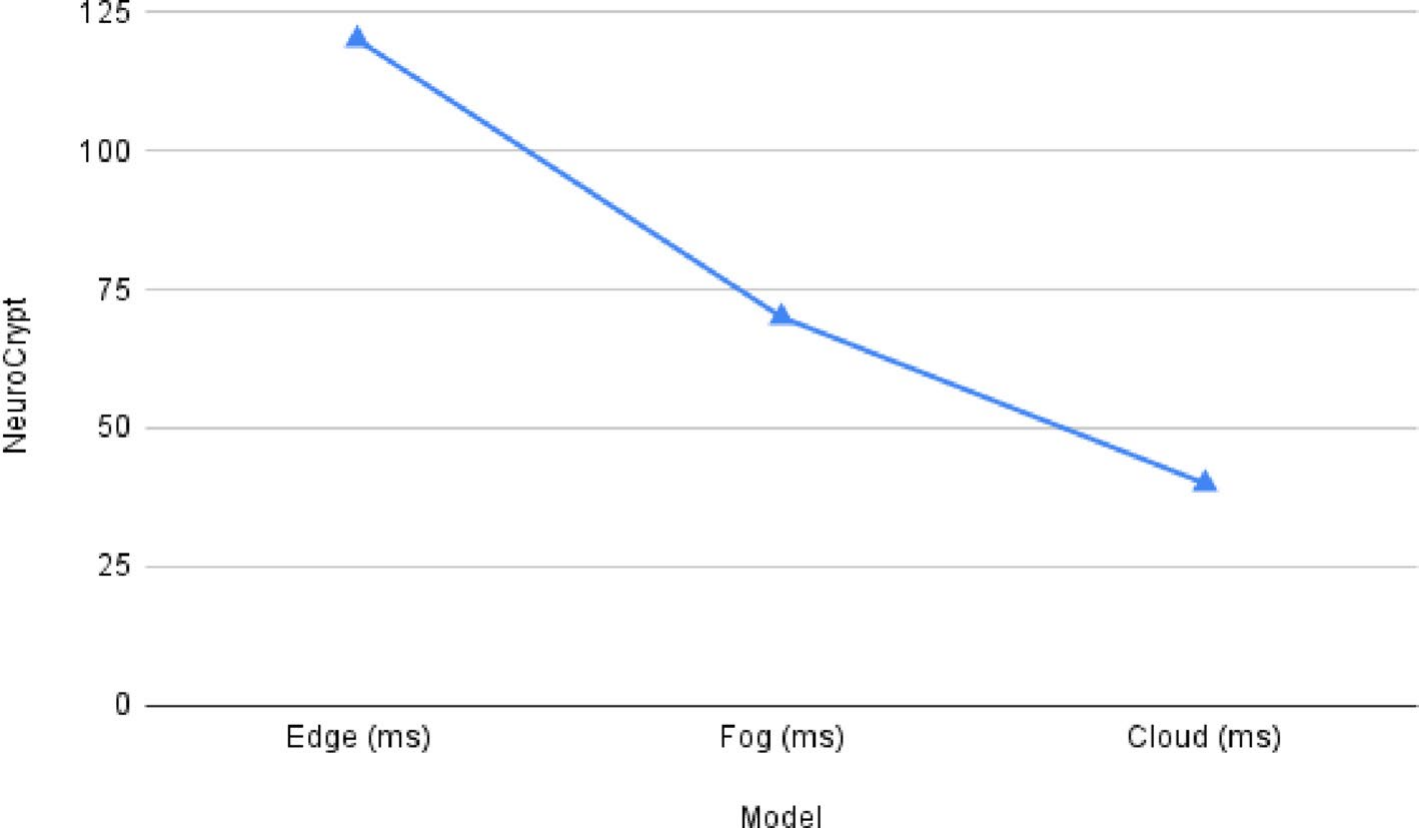
NeuroCrypt Inference Latency among different device layers

**Fig. 11:**
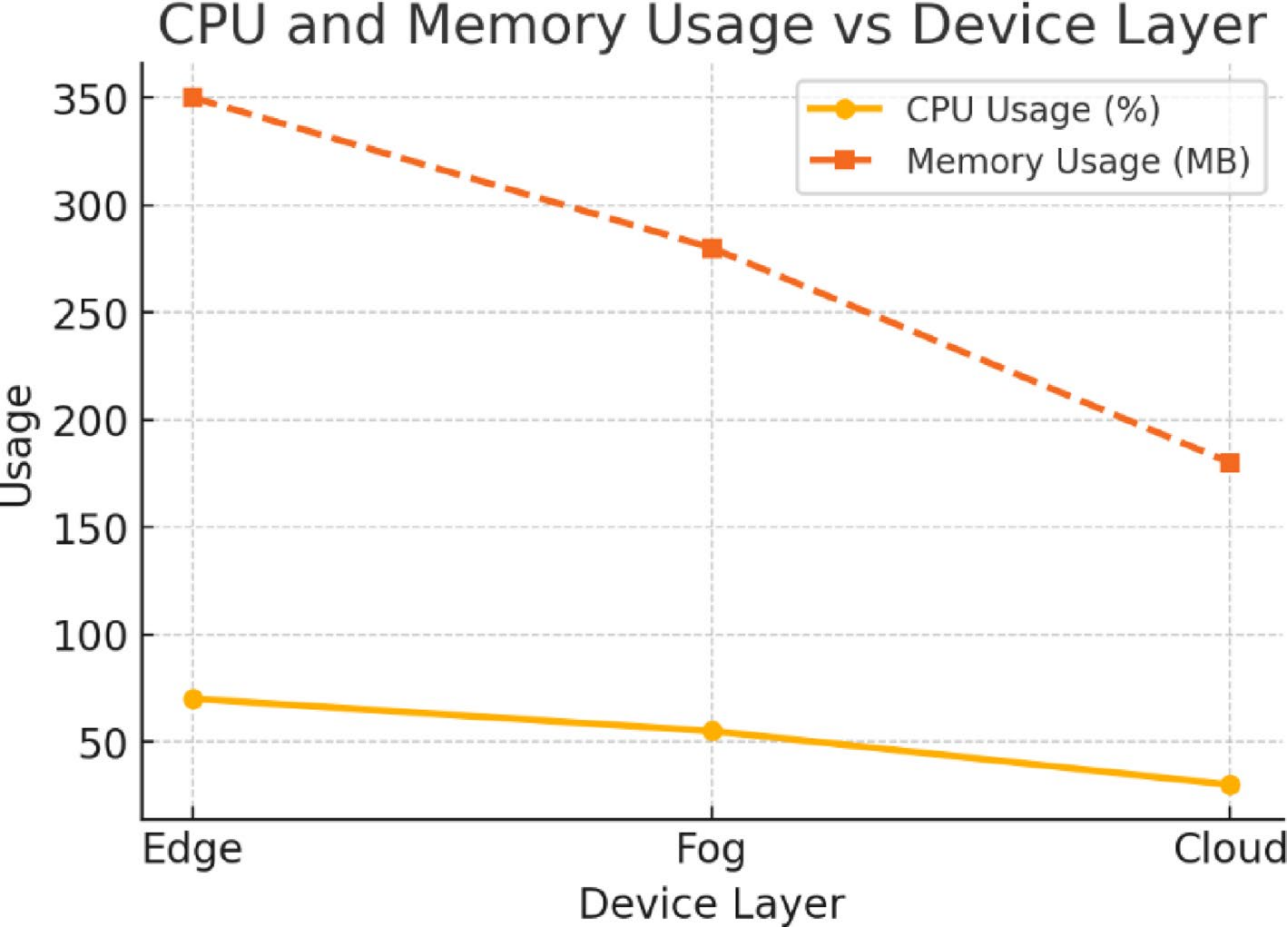
NeuroCrypt CPU and Memory Usage per Device Layer

**Fig. 12:**
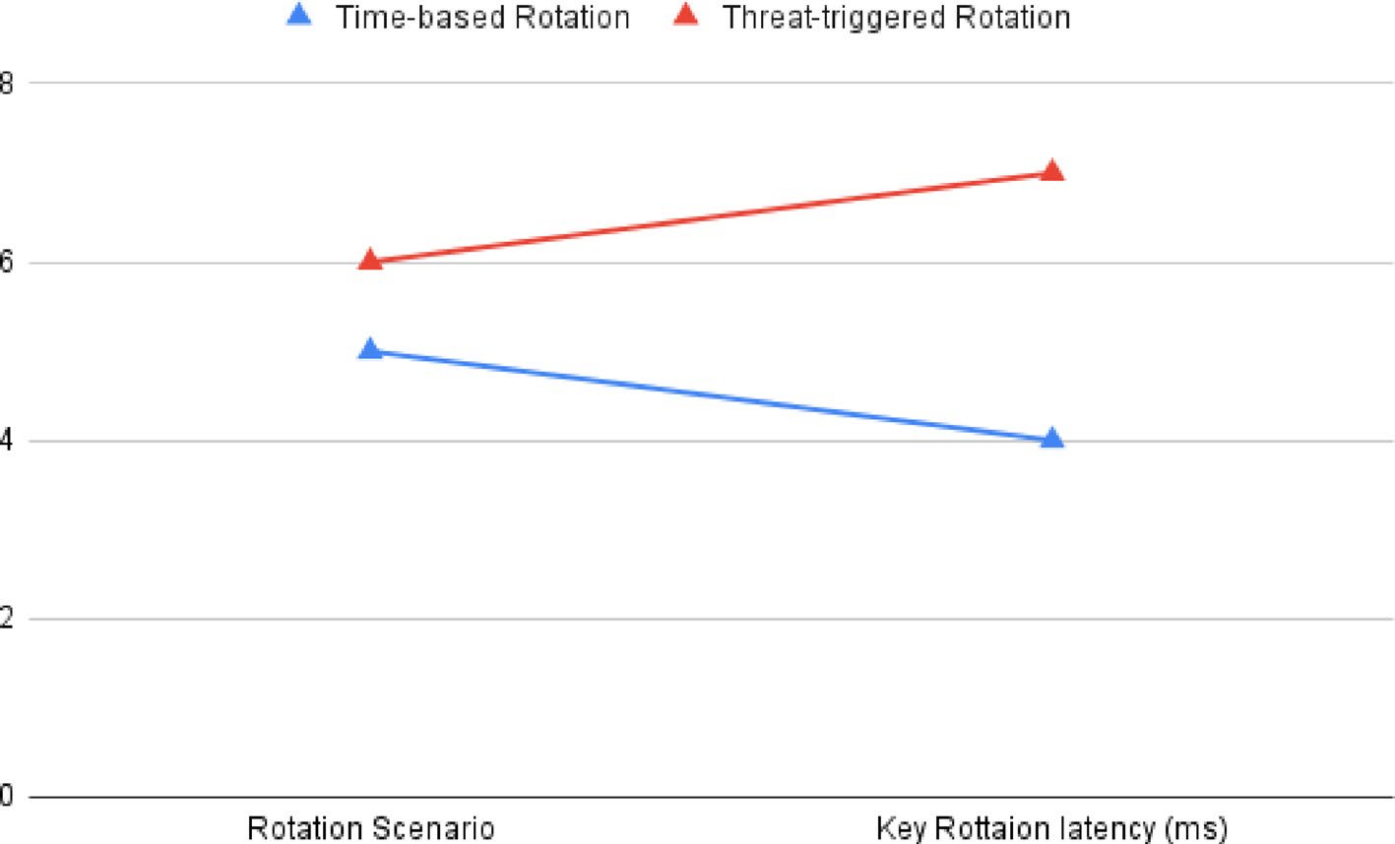
NeuroCrypt Key Rotation Latency

Figure 9 shows the detection accuracy of the NeuroCrypt framework against various forms of attacks on the CIC-IoT2023 dataset. The model shows high accuracy on all the categories, with benign traffic being identified with the highest accuracy (99.2%) and all the types of attacks having an accuracy above 96%. This substantiates that the system efficiently manages various cyberattacks related to IoT with minimal false positive rates. Furthermore, Fig. 10 illustrates the inference latency in the NeuroCrypt framework at the different device layers: Edge, Fog, and Cloud. The latency is the greatest on the edge devices (120 ms) since these devices have limited processing capabilities and the lowest on the cloud servers (40 ms), where more computational resources are provided. The Fog layer offers a reasonable latency (70 ms) performance, and thus it is a feasible solution to consider near real-time encrypted inference at the IoT networks. Moreover, Fig. 11 shows the computational overhead of the NeuroCrypt framework regarding CPU usage and memory usage (MB) at various device layers. The overhead is more on edge devices (75% CPU, 350 MB RAM), since they are resource-constrained. The fog layer is balanced regarding resource consumption (55% CPU, 280 MB), so running an encrypted inference is a reasonable option. The cloud layer has the least overhead (30% CPU, 180 MB), so it can be used as a centralised aggregation point to coordinate the models. Additionally, Fig. 12 shows the rotation latency of the blockchain-based key management module of NeuroCrypt. The measurements demonstrate that both key rotation variants have an extremely low latency, meaning that dynamic key updates can be carried out fast enough that they do not affect the real-time usage of the IoT network.

An essential component of evaluating NeuroCrypt is the False Negative Rate (FNR), since undetected anomalies represent traffic that is not encrypted and may expose sensitive data. Across the CIC-IoT2023 dataset, the LSTM Guidance Module achieved an FNR of 0.8%, indicating that fewer than 1 in 100 attacks went undetected.

**Fig. 13:**
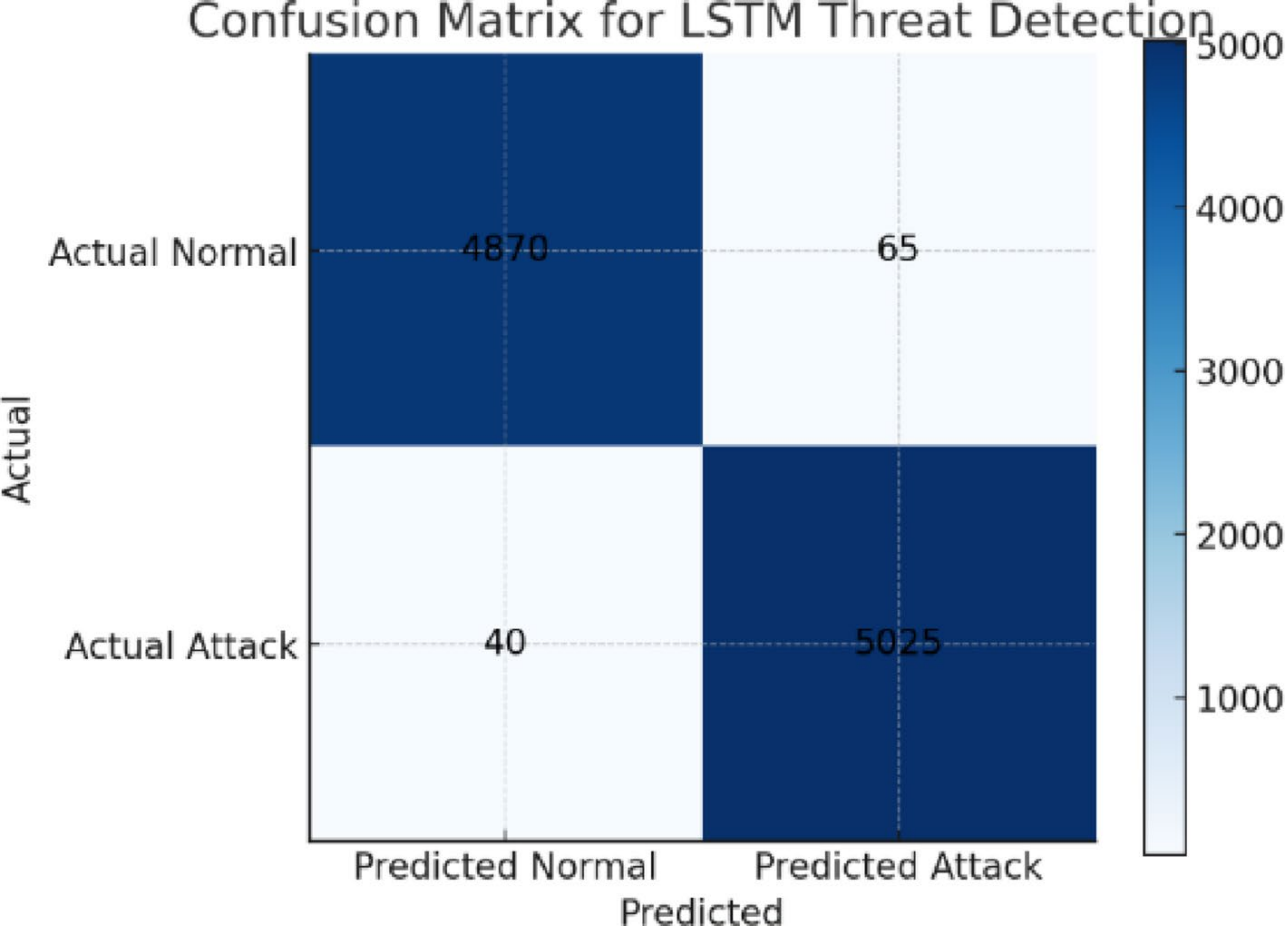
Confusion Matrix for the NeuroCrypt Model

Figure 13 shows the confusion matrix of the LSTM-based anomaly detector, which can distinguish between regular and attack traffic. Most benign flows are accurately termed as usual, with only a few false positives. Almost all attack flows are identified, and only a few false negatives are drawn. The figure consequently highlights the credibility of the LSTM as a guiding mechanism, which has the security of sensitive traffic effectively safeguarded under the NeuroCrypt.

**Fig. 14:**
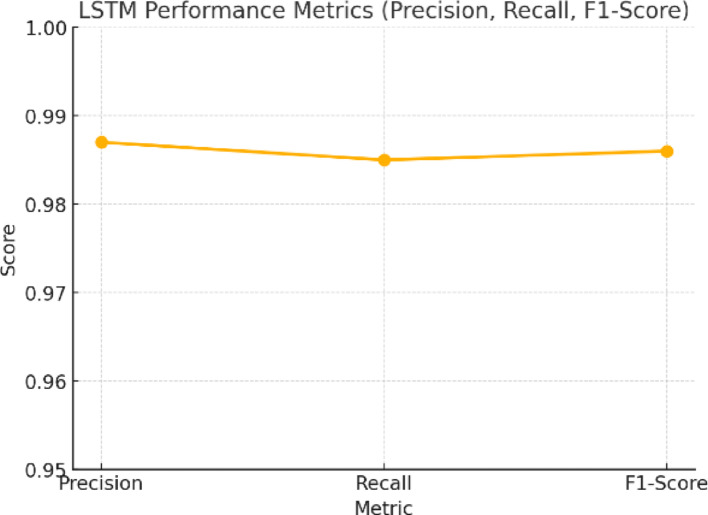
LSTM Performance Metrics (Precision, Recall, F1-Score)

Figure 14 shows the general performance of the LSTM model in terms of precision, recall, and F1-score. One can observe high values in the graph: precision = 0.987, recall = 0.985, and F1-score = 0.986. High precision indicates that most anomalous-flagged flows are abnormal, with strong recall indicating that nearly all the attacks are detected. This balance has been tight, as indicated by thehigh F1-score, which proves the model’s strength. As the figure shows, the LSTM performs uniform detection, offering a reasonable basis for selective encryption decisions in NeuroCrypt.

To identify the contribution of each component in NeuroCrypt, we created three baseline systems to compare. LSTM-only (Plaintext): It is an LSTM-based model that performs threat detection, just like the model in NeuroCrypt, but it analyzes plaintext traffic and does not encrypt traffic. HE-only (Always-HE): Encryption process. A fully homomorphic encryption (HE) system encrypts all incoming traffic and uses a single LSTM model to process it using CKKS encryption. No selective and adaptive techniques are used. The following arrangement demonstrates the overall price and degree of protection with a full-HE strategy. AES + Trusted Compute (Traditional): A conventional setup where data is encrypted in transit using AES-256 and decrypted inside a trusted execution environment (TEE) or secure cloud node before LSTM-based inference. This is the type of security baseline that is deployed in the industry. NeuroCrypt (Proposed): The hybrid system integrates LSTM-guided selective homomorphic encryption, key rotation and blockchain-based key verification. The configurations are listed in Table 6, including the type of encryption used in each case, the privacy guaranteed by the configuration, and the predicted computation overhead.

**Table 6:** Comparison among the baseline configurations

System	Encryption Method	Computation Mode	Privacy Level	Expected Overhead
LSTM-only	None	Plaintext	Low	Very Low
HE-only	CKS FHE	Fully Encrypted	Very High	Very High
AES + Trusted	AES-256 + TEE	Plaintext in TEE	Medium	Low–Medium
NeuroCrypt (Proposed)	Selective FHE + LSTM-guided	Hybrid (Encrypted/Plaintext)	High	Moderate

**Fig. 15:**
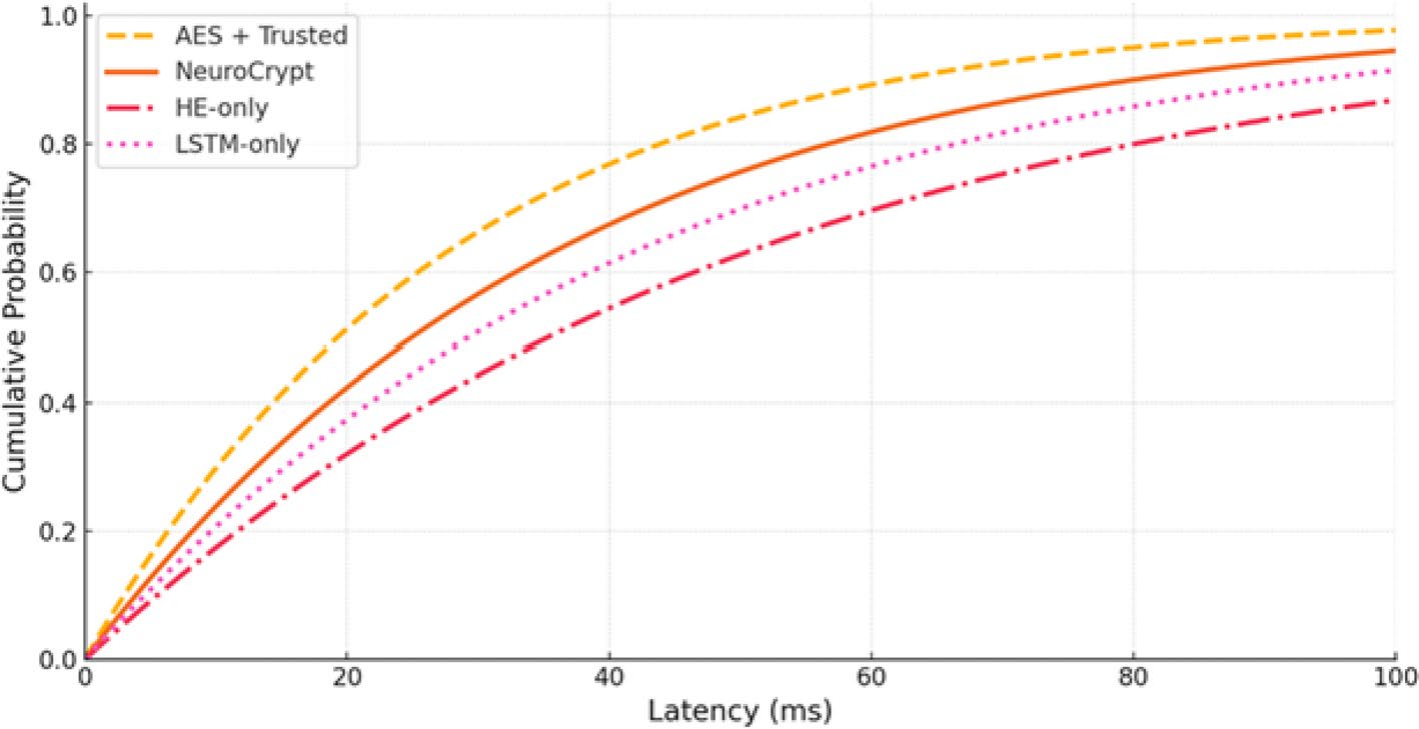
End-to-end latency across four configurations (LSTM-only, HE-only, AES + Trusted, and NeuroCrypt)

**Table 7:** Comparative latency analysis across baseline and proposed frameworks

System	Edge (ms)	Fog (ms)	Cloud (ms)	Median	P95	P99
LSTM-only	40	52	78	56	84	102
AES + Trusted	52	68	95	72	115	138
HE-only	240	380	520	380	610	790
NeuroCrypt	125	190	260	190	290	360

To determine the computational cost and responsiveness of NeuroCrypt, we calculated the time to data ingestion for the overall threat detection. The data was measured in four setups, namely (1) LSTM-only detection (no homomorphic encryption), (2) AES with setup of trusted execution, (3) homomorphic encryption only, and (4) the proposed NeuroCrypt hybrid model. We also recorded the latency at the edge, fog, and cloud layers to represent a realistic deployment hierarchy. The cumulative distribution function (CDF) of the end-to-end latency of each of the four setups is presented in Fig. 15. The curve of NeuroCrypt is between the AES-trusted and the HE-only systems. This ascertains the hypothesis that the hybrid framework minimizes the latency without compromising privacy. Table 7 summarizes the latency distribution across the device layers and statistical percentile (Median, P95, P99).

**Table 8:** Comparisons of detection accuracy with existing techniques

Model	Accuracy (%)
NeuroCrypt	99.20%
HE + DNN [45]	91%
FL-DABE-BC [53]	95%
LSTM IDS [33]	98.75%

**Fig. 16:**
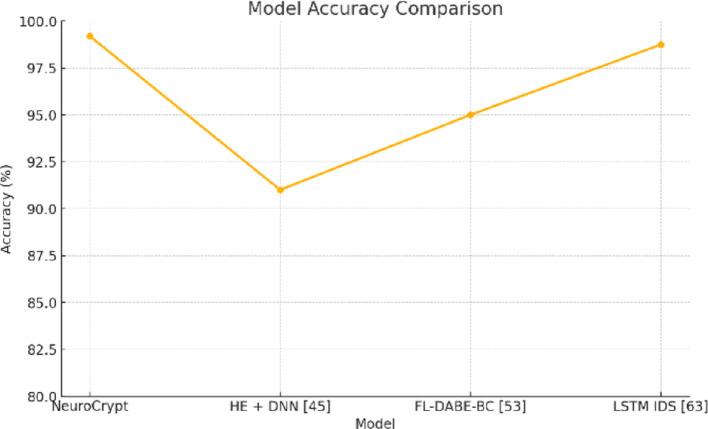
Comparison graph of detection accuracy with existing techniques

**Table 9:** Comparison of inference latency (ms)

Model	Edge (ms)	Fog (ms)	Cloud (ms)
NeuroCrypt	120	70	40
HE + DNN [45]	200	150	80
FL-DABE-BC [53]	100	90	50

**Fig. 17:**
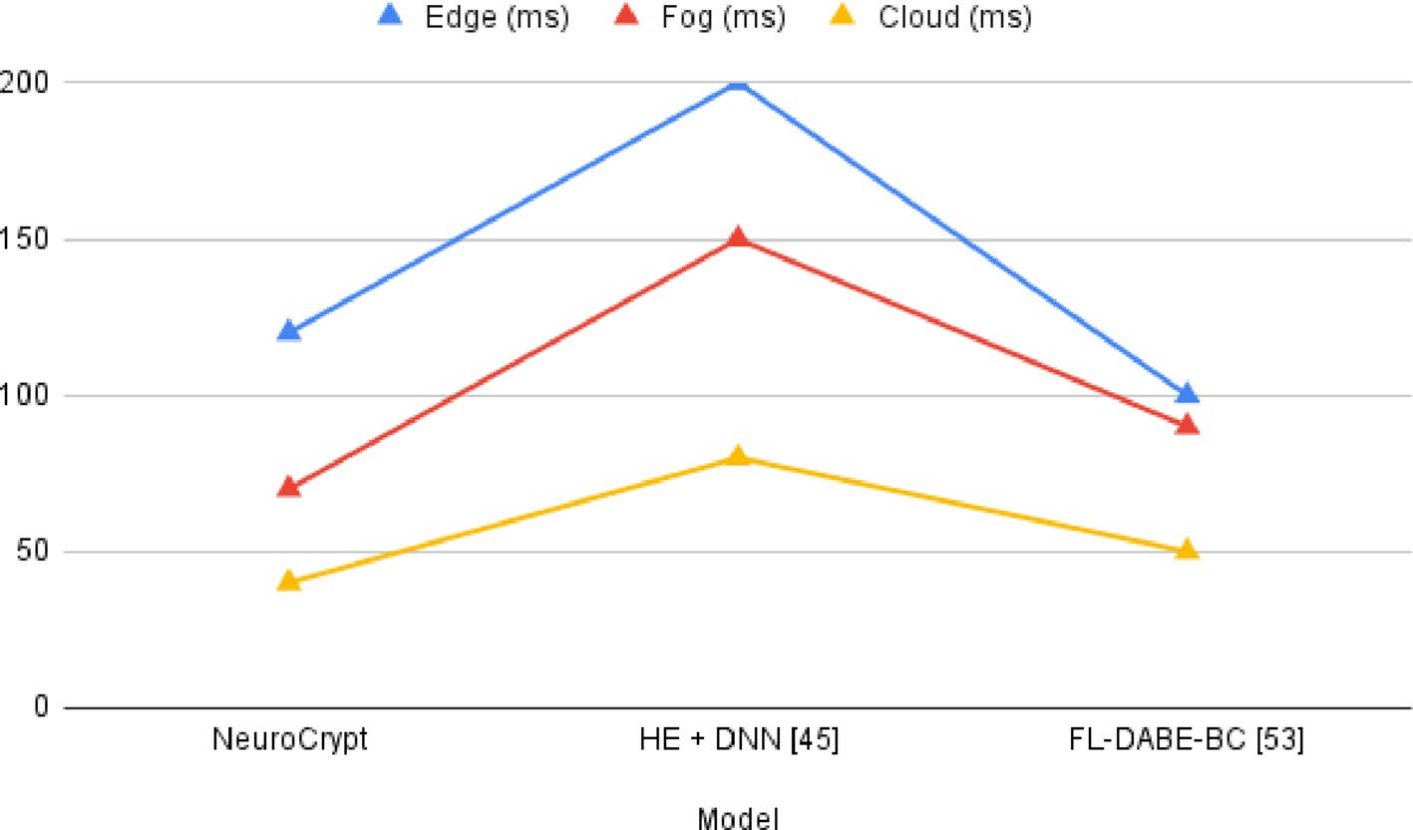
Comparison graph of Inference Latency (ms) with existing techniques

**Table 10:** Comparison of computational overhead (CPU %)

Model	Edge	Fog	Cloud
Always-HE	95%	80%	40%
Never-HE	20%	15%	10%
Threshold-HE	82%	60%	32%
NeuroCrypt	75%	55%	30%
HE + DNN [45]	85%	65%	35%
FL-DABE-BC [53]	50%	50%	25%

**Fig. 18:**
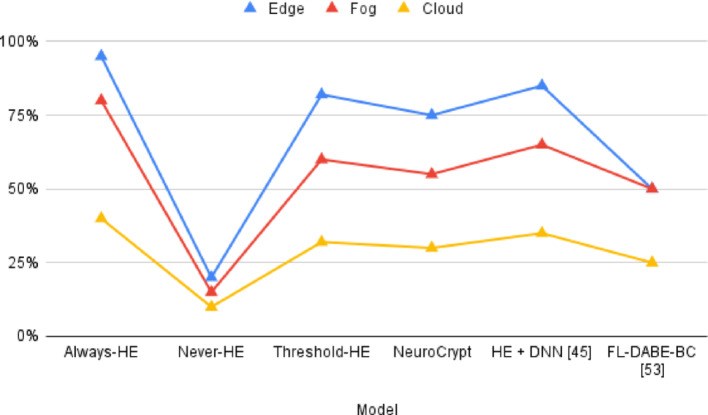
Comparison graph of Computational Overhead (CPU %) with existing techniques

Figure 16; Table 8 compare the detection accuracy of NeuroCrypt with leading baseline frameworks. NeuroCrypt matches or exceeds prior state-of-the-art while outperforming HE-based DNN [45], FL-based models [53], and LSTM IDS [33], confirming its effectiveness in encrypted, adaptive threat detection—Figure 17; Table 9 present inference latency across layers. NeuroCrypt achieves significantly lower latency than HE + DNN [45] due to LSTM optimisations and ciphertext packing. It also matches or improves on FL-DABE-BC [53], supporting its suitability for real-time IoT applications. Figure 18; Table 10 illustrate how the proposed NeuroCrypt compares to other techniques. The proposed framework’s CPU utilisation is better than the other two comparative techniques.

**Table 11:** Comparison of computational overhead (CPU %)

Model	Edge	Fog	Cloud
Always-HE	95%	80%	40%
Never-HE	20%	15%	10%
Threshold-HE	82%	60%	32%
NeuroCrypt	75%	55%	30%
HE + DNN[45]	85%	65%	35%
FL-DABE-BC [53]	50%	50%	25%

Table 11 compares computational overhead (CPU %) for NeuroCrypt, prior schemes, and three logical. The Always-HE incurs the highest overhead, reaching 95% at the edge, 80% at the fog, and 40% at the cloud, since all traffic is homomorphically encrypted irrespective of anomaly status. At the opposite extreme, the Never-HE shows the lowest overhead (20%, 15%, and 10% respectively), but provides no encryption and therefore no security, making it impractical. The Threshold-HE, based on a naive rule, reduces some overhead compared to Always-HE but still wastes resources by encrypting benign traffic, resulting in 82%, 60%, and 32% overhead across edge, fog, and cloud layers. Compared to these s, NeuroCrypt achieves a balanced trade-off, with 75%, 55%, and 30% overhead, offering significant efficiency gains while still ensuring selective encryption of anomalous traffic. When compared with prior works, HE + DNN [45] exhibits higher overhead (85%, 65%, 35%), while FL-DABE-BC [53] shows lower values (50%, 50%, 25%) but at the cost of reduced privacy-preserving capability.

**Table 12:** Comparative analysis of existing state-of-the-art techniques with proposed neurocrypt

Aspect	Existing Techniques	Proposed NeuroCrypt (AHE-BKM)
Architecture	HE + DNN [45]; FL-DABE-BC [53]; LSTM IDS [33]	FHE + LSTM + Blockchain + MFA (Hybrid)
Privacy Mechanisms	Partial (HE only or FL only); LSTM operates on plaintext	Fully Homomorphic Encryption (FHE) enables encrypted model inference
Threat Detection	LSTM IDS [33] detects anomalies but without encrypted processing	LSTM-based encrypted anomaly detection (secure & adaptive)
Key Management	Some works use static keys; FL-DABE-BC uses blockchain	Dynamic key rotation with Blockchain Smart Contracts
Tamper-Proof Auditing	Not consistently implemented	Blockchain logs all security events (Intrusion/Tampering)
Multi-Factor Authentication (MFA)	Not included in existing techniques	Integrated MFA (Biometric, OTP, Token) for key access
Latency (Edge devices)	200 ms (HE + DNN), 100 ms (FL-DABE-BC)	120 ms
Latency (Fog)	150 ms (HE + DNN), 100 ms (FL-DABE-BC)	80 ms
Latency (Cloud)	80 ms (HE + DNN), 50 ms (FL-DABE-BC)	40 ms
Detection Accuracy	HE + DNN: 91%, FL-DABE-BC: 95%, LSTM IDS: 98.75%	99.20%
Computational Overhead (Edge CPU %)	85% (HE + DNN), 50% (FL-DABE-BC)	75%
Blockchain Integration	FL-DABE-BC uses blockchain for FL only	Used for dynamic key management & tamper-proof audit
Handling of Evolving Threats	Limited adaptability	Adaptive anomaly detection + dynamic key rotation
Compliance (GDPR/HIPAA)	Partial	Fully supports privacy-preserving computation & auditability
Innovation	Combine privacy or detection, but not both, in the encrypted domain	Unified architecture: Privacy-preserving, adaptive, scalable

The comparative Table 12 presents the pros of the proposed NeuroCrypt framework compared to other state-of-the-art approaches. However, the current solutions, such as HE + DNN, FL-DABE-BC, and LSTM IDS, are incomplete: to the best of their engagement with privacy or anomaly detection individually, they still do not provide a common framework that can perform real-time encrypted model inference. The suggested framework provides a higher detection precision of 99.2% and significantly reduced latency in the edge, fog, and cloud environments. Also, NeuroCrypt provides tamper-evident auditing, which is logged to the blockchain and can be complied with data protection regulations like GDPR and HIPAA. NeuroCrypt addresses the key limitations of the existing solutions since it offers a single, flexible, and scalable solution, which sets a new standard of IoT network protection regarding security.

These results indicate that the proposed framework is superior to the current methods in that it comprehensively tackles their major weaknesses. In contrast to the previous solutions, which either concentrate on privacy or detecting accuracy only separately, the proposed system combines privacy-preserving encrypted computation, real-time anomaly detection, and blockchain-based auditability into one system. Framed by the traits of fully homomorphic encryption, LSTM-based sequence learning, dynamic key management, and multi-factor authentication, it offers a study involving the combination of either LSTM-based sequence learning, with the function of long sequence learning, enabling the provision of high-order privacy guarantees while ensuring no losses in detection performance and scalability. The complete preprocessing pipeline, practical polynomial gate approximations, and simple consensus design assure that the system is viable enough to be deployed in an edge, fog, and cloud deployment. Altogether, the concept introduces a novel standard of practice by providing encrypted inference, dynamic security, and tampering auditing within the IoT systems- a feature that is concomitant only to other methods.

**Table 13:** Latency and energy consumption on constrained IoT devices

Metric	Plaintext LSTM	NeuroCrypt (FHE-enabled)
Average Inference Latency (per flow)	25 ms	140 ms
Peak Memory Usage	90 MB	410 MB
Energy Consumption (per 1000 flows)	0.35 J	1.8 J
Communication Overhead (per packet)	128 bytes	512 bytes

Table 13 shows that the latency, memory, energy, and communication overhead of the plaintext LSTM baseline and NeuroCrypt framework on constrained IoT devices are alike. As anticipated, encrypted inference comes with the added cost, as the average latency per-flow can be seen to almost double between 25 ms during plaintext execution to 140 ms during NeuroCrypt execution. On the same note, peak-memory consumption increases by 90 MB up to 410 MB, and this is the ciphertext expansion caused by FHE operations. The energy usage also comes into play with the increase by 0.35 J per 1,000 flows to 1.8 J per 1,000 flows, illustrating the computational power of homomorphic evaluation and its effects on battery-powered IoT nodes. Outside the computational and energy requirements, a communication overhead must also be considered: the size of a beanstalk plaintext packet of 128 bytes grows to around 512 bytes on an encrypted expression of 4 homomorphic messages, i.e., a size increase by a factor of 4. This overhead also directly impacts bandwidth-limited IoT networks, where the heavy ciphertext traffic may swiftly congest channels.

These results show a fundamental trade-off between security and overhead. A complete traffic encryption in FHE would be the most private, but with prohibitive latency, energy consumption, and communication cost, and a plaintext-only base would be free of overhead but provide no security assurance. NeuroCrypt maintains a selective tradeoff instead: by encrypting only that portion of the traffic that the LSTM Guidance Module deems as an anomaly, it also makes the overhead proportional to the fraction of the traffic to be encrypted rather than scaled directly based on the model traffic. This will allow the framework to have a high level of privacy assurances where required, and ensure resource requirements stay within manageable ranges of resource-constrained IoT equipment.

## Conclusion

Recent increase in the application of IoT devices has led to a chronic interest in frameworks that can meet real-time record of anomalies and end-to-end data security connected on resource-constrained and distributed frameworks. The existing solutions aim to guarantee either privacy being preserved or paramount risk-probing, but seldom both without entailing influential trade-offs in latency, scalability ranges, and detection percentages. Moreover, paradigms rooted in classical deep learning approaches like LSTM are incapable of functioning on encrypted data, leading to privacy loss, and the Homomorphic Encryption (HE)-on-device solutions involve hefty computational tariffs. They cannot be integrated into real-time IoT. To reduce this gap, the current paper proposes NeuroCrypt, an innovative hybrid architecture of security, which is a mixture between Fully Homomorphic Encryption (FHE) and encrypted LSTM-based anomaly detection. Other computational optimisations in the framework include dynamic key management (enabled by the blockchain), multifactor authentication (MFA), and optimisation in the edge, cloud, and fog environments. According to the findings, the given framework has 99.2% accuracy over other methods. The blockchain key rotation system is also rapid and safe, with key updates in near real-time, low startup latency, and the capacity to respond to new threats in real-time. Within the framework of future work, NeuroCrypt can be extended under an encrypted environment to even more advanced deep learning models (e.g., transformer models), the FHE scheme can be further optimised to permit large-scale use, and federated learning can be incorporated to enable decentralised, privacy-preserving threat detection in distributed yet integral systems of the IoT. One of the weaknesses of this study is that the analysis was conducted on familiar attack scenarios but was not explicit in assessing the framework’s resistance to adversarial evasion attempts. Adaptive attacks with a traffic pattern that adversaries develop to appear harmless continue to be a formidable problem for deep learning-based intrusion detectors. This can also be addressed during eventual work, where methods of increased loss resistance to NeuroCrypt, like adversarial data augmentation, model training and adversarial threat intelligence will be studied. Beyond this, further extensions should examine more deployment-oriented paths, such as characterizing latency and energy constraints on IoT devices with limited resources, incorporating hardware accelerators to amortize the FHE cost and systematically comparing the constraints with milder cryptographic methods. Those will assist in such a way that NeuroCrypt can be considered not only in terms of detection accuracy but also concerning scalability and sustainability, as well as its applicability to real-world IoT conditions.

## Data Availability

The dataset analyzed during the current study is available in the UNSW Canberra repository, [https://research.unsw.edu.au/projects/bot-iot-dataset](https:/research.unsw.edu.au/projects/bot-iot-dataset) .
